# Critical Success Factors Influencing the Acceptance of a Casemix-Based Hospital Information System: Cross-Sectional Study

**DOI:** 10.2196/74226

**Published:** 2025-09-29

**Authors:** Noor Khairiyah Mustafa, Roszita Ibrahim, Syed Mohamed Aljunid, Azimatun Noor Aizuddin, Zainudin Awang

**Affiliations:** 1 Department of Public Health Medicine Faculty of Medicine Universiti Kebangsaan Malaysia Cheras, Federal Territory of Kuala Lumpur Malaysia; 2 Ministry of Health Malaysia Putrajaya, Federal Territory of Putrajaya Malaysia; 3 Department of Public Health and Community Medicine, School of Medicine IMU University Bukit Jalil, Federal Territory of Kuala Lumpur Malaysia; 4 International Casemix Centre (ITCC) Hospital Canselor Tuanku Mukhriz Cheras, Federal Territory of Kuala Lumpur Malaysia; 5 School of Management Sciences, Faculty of Business and Management, Gong Badak Campus Universiti Sultan Zainal Abidin Kuala Terengganu Malaysia

**Keywords:** healthcare information technologies, technology acceptance, structural equation modeling, direct effects, higher-ranked predictors, lower-ranked predictors

## Abstract

**Background:**

The Ministry of Health Malaysia integrated the Casemix System into the Total Hospital Information System (THIS) to improve care delivery, resource efficiency, and cost-effectiveness. Casemix, a patient classification tool, supports clinical documentation, hospital financing, and management by grouping patients according to diagnoses and resource use. Within THIS, it enables automated coding, streamlined workflows, and better hospital performance. Its success, however, relies on acceptance by medical doctors who ensure accurate documentation and coding. Despite its importance, limited empirical research has examined factors influencing Casemix acceptance in Malaysia’s hospital information system context. Understanding these factors is critical for effective implementation and sustained use.

**Objective:**

This study aims to investigate the interrelationships between critical success factors namely system quality (SY), information quality (IQ), service quality (SQ), organizational characteristic (ORG), perceived ease of use (PEOU), perceived usefulness (PU), and intention to use (ITU) on user acceptance of the Casemix system in hospitals equipped with THIS.

**Methods:**

This study used a cross-sectional design, using a self-administered digital questionnaire that was developed by adopting and adapting previously validated instruments, grounded in the Human-Organization-Technology Fit and Technology Acceptance Model (TAM) frameworks. The instrument underwent rigorous validation and reliability procedures, including content and criterion validation through expert review, exploratory factor analysis to assess item appropriateness, and confirmatory factor analysis to establish construct, convergent, and discriminant validity. Proportionate stratified random sampling was used to ensure equitable representation of medical doctors across 5 Ministry of Health hospitals, each representing 1 of Malaysia’s geographical zones. The minimum required sample size of 375 was proportionally distributed across 4 categories of medical doctors within these hospitals. Based on structural equation modeling standards, a total of 343 valid responses were obtained, yielding a response rate of 91.5%. Path analysis was conducted using covariance-based structural equation modeling with SPSS Amos (version 24.0; IBM Corp) to assess the direct relationships among the constructs in this study.

**Results:**

Path analysis revealed that SY (β=–0.262, *P*=.043) and IQ (β=0.307, *P*=.01) significantly influenced PEOU. PEOU (β=0.105, *P*=.02) and PU (β=0.580, *P*<.001) significantly influenced ITU, which strongly predicted user acceptance (β=0.788, *P*<.001). PEOU did not substantially impact PU (β=0.086, *P*=.07), nor did SQ (β=0.146, *P*=.19) and ORG (β=0.197, *P*=.21) significantly influence PEOU. Based on the β coefficients and statistical significance, the critical success factors were categorized into 2 groups: higher-ranked predictors (ITU, PU, IQ, and SY) and lower-ranked predictors (ORG, SQ, and PEOU). Higher-ranked predictors demonstrated statistically significant relationships and relatively stronger β coefficients.

**Conclusions:**

This study offers empirical insights into key factors influencing Casemix system acceptance and informs strategies to support its successful implementation in THIS-equipped hospitals. The findings also contribute to addressing current research gaps and guiding future evaluations of health care information systems.

## Introduction

### Background

Worldwide, the adoption of Hospital Information Systems (HIS) and Casemix systems has been recognized as a critical strategy for enhancing health care quality, optimizing resource allocation, and controlling costs in line with international health system strengthening agendas and Sustainable Development Goal 3: Good Health and Well-Being [1-8]. HIS comprising hardware, software, processes, and governance structures enables integrated management of clinical, administrative, and financial data to support efficient, high-quality patient care [9-14]. Within this broader category, the Total Hospital Information System (THIS) represents a fully integrated, paperless information and communications technology solution incorporating modules such as the CIS (Clinical Information System), NIS (Nursing Information System), LIS (Laboratory Information System), RIS (Radiology Information System), PIS (Pharmacy Information System), and PACS (Picture Archiving and Communication System) [6-8,15].

Casemix systems, originally developed in Australia and popularized through DRGs (Diagnosis-Related Groups) in the United States, classify patient episodes based on clinical and resource-use characteristics, enabling more transparent health care financing, performance monitoring, and equitable resource allocation [16-23]. These systems are widely deployed in high-income countries and increasingly in upper middle-income countries (UMICs) and lower middle-income countries (LMICs), although implementation in UMICs and LMICs often faces barriers such as inadequate infrastructure, limited technical capacity, and resistance to change [24-30]. Nevertheless, successful UMICs and LMICs experiences such as Indonesia’s national rollout of the INACBGs (Indonesian Case-Based Groups) demonstrate the feasibility and value of context-specific implementation strategies [25,31].

In Malaysia, the Ministry of Health (MOH) began HIS digitalization in 2001 with THIS at Hospital Selayang, followed by nationwide rollouts of MalaysianDRG (Malaysian Diagnosis-Related Group) applications to support Casemix costing and performance monitoring [6-8,32-37]. The latest iteration, MyCMX 3.0, launched in 2025, delivers clinical, costing, and pay-for-performance data to hospital managers [32,33]. However, Casemix remains a standalone system in MOH hospitals, lacking full interoperability with THIS, IHIS (Intermediate Hospital Information System), or Basic Hospital Information System platforms [32,33]. This separation creates fragmented workflows, duplicate data entry, and limited real-time decision support, reducing the system’s overall impact.

While HIS and Casemix adoption have been extensively studied in high-income settings, such as the United States, Western Europe, Australia, and Eastern Europe [22,23,27-30], research in UMIC and LMIC contexts, especially on the integration of Casemix into national HIS, remains limited. Malaysian studies have primarily examined HIS adoption in general terms [9-11,38], with few addressing the complex interplay of technological, organizational, and human factors that determine successful integration and user acceptance (UA) [9-12,24-26,32,33,38]. Moreover, existing literature often isolates individual-level behavioral constructs (eg, Technology Acceptance Model [TAM] from broader system and organizational determinants (eg, Human, Organization, and Technology Fit Evaluation Framework [HOT-Fit] leaving a gap in comprehensive adoption frameworks [13,14,29,30,39-41].

This study addresses these gaps by developing and validating the Acceptance of Casemix in Total Hospital Information System (ACT) model, integrating TAM and HOT-Fit to capture both behavioral (perceived ease of use [PEOU], perceived usefulness [PU], intention to use [ITU], and UA) and organizational-technological (system quality [SY], information quality [IQ], service quality [SQ], and organizational characteristic [ORG]) determinants of Casemix adoption in THIS-equipped MOH hospitals. By situating Malaysia’s experience within the global digital health landscape, this research provides new theoretical insights into multidimensional adoption factors and offers practical recommendations for HIS-Casemix integration strategies in UMICs and LMICs pursuing health care financing reform.

### Underpinning Theories and Conceptual Framework

This study uses an integrated framework combining the TAM and the HOT-Fit model to evaluate Casemix acceptance within Malaysia’s THIS. TAM, widely used in health care IT (HIT), explains individual-level adoption through PEOU, PU, ITU, and UA [9,39,41-43]. These constructs capture doctors’ behavioral intentions but overlook organizational and technological factors. HOT-Fit complements TAM by incorporating SY, IQ, SQ, and ORG, which influence alignment with workflows, infrastructure, and support [13,29,30]. As a multidimensional digital intervention, Casemix requires both UA (explained by TAM) and organizational-technological readiness (explained by HOT-Fit). Evidence shows HIS adoption succeeds only when human, organizational, and technical factors are addressed together. Thus, integrating TAM and HOT-Fit provides a comprehensive lens for evaluating Casemix adoption, advancing existing models by framing adoption as a sociotechnical outcome, particularly relevant in UMIC and LMIC contexts.

### Independent Variables

#### About SY

SY refers to the desirable attributes of the Casemix system and THIS that ensure operational efficiency and reliability. Key characteristics include usability, system reliability, response time, integration capacity, security features, and maintainability. These attributes affect users’ overall experience and trust in the system’s performance [13,30,44-50].

#### About IQ

IQ, in the context of HIT, addresses the integrity, usefulness, and reliability of the data produced and exchanged within systems such as the Casemix and HIS. This includes completeness, accuracy, relevance, timeliness, and clarity of information attributes essential for clinical decision-making and administrative processes [13,30,44-46,50].

#### About SQ

SQ is encompassing IT responsiveness, troubleshooting, and user training, is a key determinant of healthcare professionals’ satisfaction and technology adoption. Core dimensions include assurance, empathy, reliability, and tangible support [13,30,44,50-52] on electronic medical records and HIS shows that strong IT support enhances user confidence and sustained system use [13,30,44,50-52].

#### About ORG

ORGs are contextual factors influencing HIT adoption, including structure (hierarchy, communication, and task distribution) and environment (leadership, communication, and regulatory readiness). Evidence shows that supportive management, adaptive cultures, and adequate resources promote higher system acceptance and integration [13,30,50,53-55]. These factors shape resource allocation, adoption decisions, and user behavior.

#### About PEOU

PEOU reflects users’ belief that interacting with the system will be free of effort. It emphasizes intuitive design, clarity of navigation, and minimal complexity during interaction. As a fundamental element of TAM, PEOU significantly shapes attitudes and behavioral intentions toward system use [14,39,41,56-59].

#### About PU

PU is the degree to which users believe that using the Casemix and THIS systems will enhance their job performance. It captures improvements in clinical efficiency, workflow integration, and the system’s role in supporting decision-making and health care delivery. PU is a core construct in TAM and a known predictor of behavioral intention [14,39,41,56-59].

#### About ITU

ITU refers to the motivational state that drives users’ decisions to adopt and continue using a given technology. ITU is often shaped by PU and PEOU and serves as a proximal predictor of actual system UA. In the health care setting, it represents professionals’ readiness to integrate HIS/Casemix tools into their clinical or administrative tasks [14,39,41,56-59].

### Dependent Variable: About UA

UA reflects health care providers’ willingness and commitment to adopt and consistently use the Casemix system within THIS. It encompasses attitudes, confidence, and satisfaction in performing tasks efficiently. High UA signifies successful implementation, sustained use, and improved health care delivery and institutional efficiency [14,42,57,60,61].

**Figure 1:**
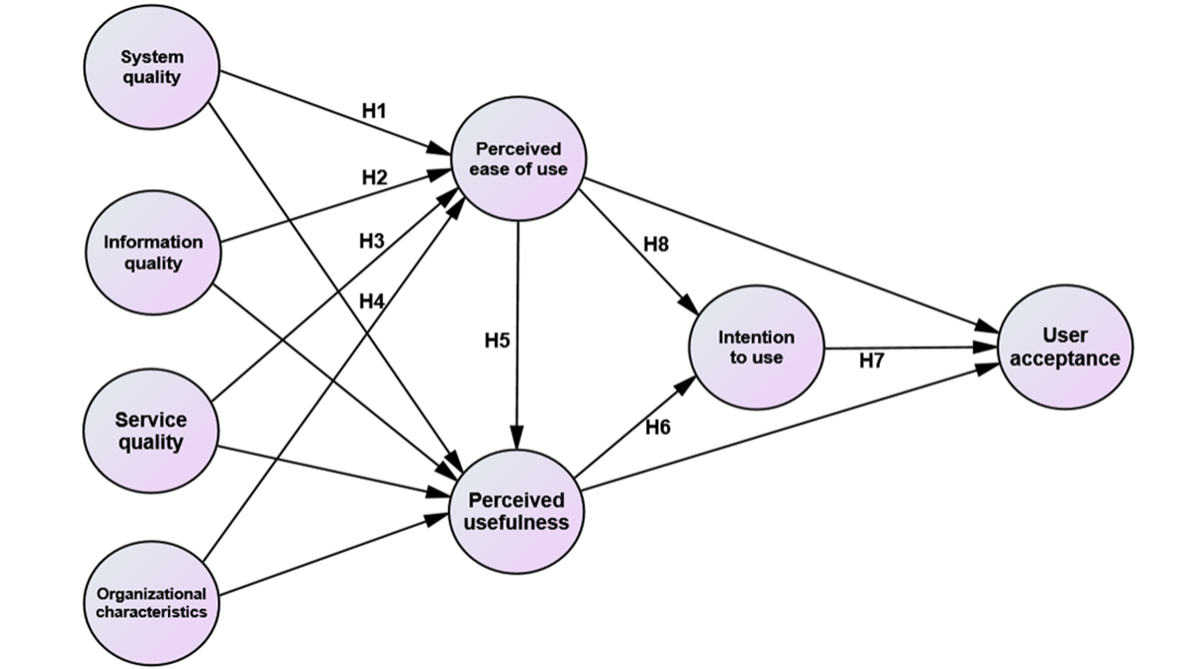
The conceptual framework. H: hypothesis.

Therefore, a validated multi-item questionnaire in this study, adapted from prior studies, was used to measure the constructs [9,13,29,30,39,41,43]. The structural model was tested using covariance-based structural equation modeling (CB-SEM) to identify critical success factors (CSFs) influencing UA through direct effect analysis. While TAM and HOT-Fit have been applied separately in health IT, this study is among the first to integrate them for examining Casemix acceptance in THIS context. With MOH Malaysia’s digital transformation agenda and the strategic role of Casemix in health care financing, this study addresses a critical gap in understanding adoption within complex HIS environments marked by infrastructural variability and inconsistent integration [12,32,33,38]. Hence, 8 hypotheses (H1-H8) were proposed (Figure 1): SY (H1), IQ (H2), SQ (H3), and ORG (H4) exert a significant direct effect on PEOU; PEOU exerts a significant direct effect on PU (H5); PU to significantly affect ITU (H6); ITU has substantial direct effect on UA (H7); and PEOU to substantially affect UA (H8).

## Methods

### Study Design

This study used a quantitative, cross-sectional design, intended to elaborate on the quantitative results. This paper focuses solely on the quantitative phase, which examines the effects of 7 CSFs on the acceptance of the Casemix system among medical doctors in MOH hospitals equipped with THIS [62]. This study was conducted in two stages: (1) a pilot study (February 1-14, 2023) for instrument validation, followed by (2) the main survey (April 1 to June 30, 2023). The flowchart of this study can be observed in Multimedia Appendix 1. This paper reports specifically on the methodology, results, and interpretation of this study.

### Ethical Considerations

Ethical approval was obtained from the MOH Malaysia (NMRR ID-22-02621-DKX) and Universiti Kebangsaan Malaysia (JEP-2022-777; see Multimedia Appendix 2), with additional permissions from the Medical Development Division and participating hospital directors. Participation was voluntary, with electronic informed consent obtained, and no compensation was provided. The survey, administered via Google Forms, collected no identifiable information (including IP addresses); all responses were anonymized and assigned nontraceable IDs. Data access was restricted to the principal investigator and stored on encrypted, password-protected drives, in accordance with the Malaysian Personal Data Protection Act, the Declaration of Helsinki, and institutional guidelines. Participation was voluntary, with electronic informed consent obtained, and no compensation provided.

### Study Instrument

The self-administered questionnaire was developed in English and Bahasa Melayu/Malay using back-to-back translation. Based on HOT-Fit [13,29,30] and TAM [9,39,41-43], it comprised 59 items: demographics (8), Casemix knowledge (10), CSFs constructs (36: SY, IQ, SQ, organizational structure [STR], organizational environment [ENV], PEOU, PU, ITU), and UA (5). Sections 2-3 used a 10-point Likert scale (1=strongly disagree, 10=strongly agree) [63-66]. The instrument was pilot-tested and validated through exploratory factor analysis (EFA) and confirmatory factor analysis (CFA). Detailed operational definitions for each item appear in Multimedia Appendix 3 [1,13,14,18,29,30,33,36,37,39,41,42,44,45,47,49-51, 56,57,60,61,67,68].

### Outcome Measures

The primary outcome was UA of the Casemix system, measured by validated TAM constructs (PEOU, PU, and ITU) and HOT-Fit constructs (SY, IQ, SQ, and ORG). Feasibility and acceptability were operationalized through survey responses reflecting system use, perceived quality, and organizational support.

### Study Location and Population

This study’s population comprised 3580 registered medical doctors from 5 MOH hospitals with operational Casemix modules, purposively selected to represent mature system use and consistent Casemix reporting across Malaysia’s regions (North, South, Central/West, East Coast, and East Malaysia). For confidentiality, hospitals are anonymized as Hospital N, Hospital S, Hospital W, Hospital E, and Hospital EM. Participants included hospital directors, deputy directors (medical), consultants or specialists, medical officers, and house officers.

Eligibility requirements were (1) a valid Malaysian Medical Council license, (2) ≥3 months’ experience using THIS with Casemix, and (3) active involvement in patient management or Casemix-related decision-making. Participation was voluntary with informed consent. The Human Resource Departments and Casemix System Coordinators provided study population data. The samples used for face validation or pretesting and the pilot test shared similar characteristics with the field study population, but were excluded from the final field survey. Details of inclusion and exclusion criteria are in Multimedia Appendix 4.

### Methods of Sample Size and Sampling

Proportionate stratified random sampling was used to ensure representation across hospitals and professional strata (hospital and deputy directors representing the top management, specialists, medical officers, and house officers) [67-70]. Sample size determination was guided by SEM/CFA requirements, with recommendations ranging from 100-150 for simple models [71,72] to 250-500 for complex models [73,74], with a 5-10:1 case-to-parameter ratio or at least 100 observations per group in multigroup analysis [75-77]. Hence, this study is using a 5:1 item-to-response ratio for the 59-item instrument [75,76], and allowing a 20% nonresponse rate, the final target was 370, using the formula N=n/(1–d) [78].

### Methods of Data Collection

A total of 343 valid responses were obtained (91.5% response rate). Questionnaires were disseminated via HODs and Casemix Coordinators through professional WhatsApp groups and email. Proportionate stratified random sampling was applied using hospital-specific quotas (eg, 50 medical officers and 30 specialists at Hospital N) to ensure balanced representation. The digital survey included a participant information sheet and consent form (Multimedia Appendices 5-7, respectively), outlining objectives, key terms, and instructions. Participation was voluntary, anonymous, and confidential, with rights to withdraw guaranteed. Consent was provided electronically (“I agree” and “I disagree”), consistent with ethical standards [79]. Respondents logged in with email addresses, allowing 1 response per account; data were cleaned of duplicates, with no missing entries.

### Methods of Data Analysis

Data analysis was conducted in 2 phases: instrument validation and hypothesis testing. Statistical analyses were performed using SPSS (version 25.0; IBM Corp) for preliminary analyses and EFA, and Amos (version 24.0; IBM Corp) for CFA and CB-SEM [65,66,71,80,81].

### Phase 1: Items Exploration

Before field data analysis, the pilot study underwent EFA using principal component analysis (PCA) with varimax rotation to identify factor structures and refine the measurement model. Item retention followed strict criteria [64-66,71,80-82]: (1) Kaiser-Meyer-Olkin (KMO) measure of sampling adequacy>0.6. (2) Bartlett test of sphericity (BTOS) *P*<.05. (3) Factor loading≥0.60. (4) Eigenvalues>1.0. (5) Total variance explained (TVE) exceeding 60%. (6) Cronbach α>0.70 for internal consistency.

The pilot EFA results confirmed dimensionality and reliability, leading to the removal of 1 item, item O1, from the organizational factors construct. These refined constructs were then used in the field study.

### Phase 2: Measurement Model Assessment

The field study measurement model was assessed through CFA to establish [65,66,81,83-85]: (1) unidimensionality: confirmed by acceptable factor loadings (>0.60). (2) Construct validity: model fit was evaluated using multiple indices: absolute fit: root-mean-square error of approximation (RMSEA) between 0.05 and 1.00; incremental fit: comparative fit index (CFI), Tucker-Lewis index (TLI)≥ 0.90; and parsimonious fit (*χ*²_750_=1510.8 is 2.014, target<3.0), (3) Convergent validity: evaluated using average variance extracted (average variance explained [AVE]>0.50) and composite reliability (CR>0.70). (4) Discriminant validity: confirmed when the square root of AVE exceeded interconstruct correlations. (5) CR values above 0.70 indicated satisfactory internal consistency. (6) Normality was checked through skewness and kurtosis (acceptable range: −1.5 to +1.5). Pooled-CFA was preferred as it offered higher degrees of freedom and better model identification for constructs with fewer indicators [65,66,81,83,86]. Modification indices were reviewed to optimize model fit without compromising theoretical integrity.

### Phase 3: Structural Model Assessment (CB-SEM) and Hypothesis Testing

The validated measurement model was incorporated into the structural model, which tested the hypothesized relationships among 8 latent constructs (SY, IQ, SQ, ORG, PEOU, PU, ITU, and UA) [9,39,41-43]. CB-SEM was selected for its strength in testing theory-driven relationships [65,66,71,80,81]. Given the moderately complex SEM used in this study, several strategies were implemented to mitigate the risks of overfitting, multicollinearity, and inflated type I error rates. First, the final sample size of 343 participants was considered adequate based on established SEM guidelines, which recommend 5 to 10 participants per estimated parameter and a minimum of 200 for models involving latent constructs [71,75,76]. Second, rigorous validation of the measurement model was conducted through exploratory and confirmatory factor analyses (EFA and CFA) to ensure construct validity, structural integrity, and unidimensionality of the latent variables. Third, multicollinearity was assessed through Pearson correlation coefficients among exogenous constructs, all of which remained below the 0.85 threshold, indicating sufficient discriminant validity [65,66]. Finally, model fit was evaluated using a range of indices similar to CFA model fit indices [65,66,81,87]. Direct effects between constructs were assessed using structural path coefficients derived from the unstandardized structural model. The significance of each path (*P*<.05) determined whether the corresponding hypotheses were supported.

### Phase 4: Categorization and Stratification of CSFs

The identified CSFs were categorized and stratified based on their statistical significance and effect sizes derived from the structural model analysis using CB-SEM. Standardized β coefficients (β) and *P* values were used to determine the strength and significance of relationships between constructs. CSFs with significant effects (*P*<.05) and higher β values were classified as high-ranked predictors or CSFs, while those with nonsignificant effects (*P*>.05) or lower β values were categorized as low-ranked predictors or CSFs.

## Results

### Findings for the Pilot Study and Items Exploration

The pilot study used EFA via SPSS (version 25.0, with KMO measure of sampling adequacy and BTOS) to assess the suitability of the data for factor extraction across the constructs. The overall KMO value was 0.859 (>0.6) and BTOS was significant at *P*<.001 (aim: *P*<.05), confirming data suitability for factor extraction [65,66,71,82,88-90]. EFA was conducted on 106 pilot responses to assess item dimensionality, as recommended by several studies [64-66,71,81,88,91]. A total of 42 items were grouped into 9 components, with allocation confirmed through the rotated component matrix and scree plot [64-66,81,88]. The scree plot provided corroboration for identifying these components, and the specific allocation of items is shown in Figure S1 of the Multimedia Appendix 8. These results validate the constructs and provide insights into the underlying dimensions.

### Dimensions and Total Variance

Table S1 (Multimedia Appendix 8) shows 9 components with eigenvalues >1.0, ranging from 6.6 to 14.155. Variance explained was 33.187% (first component) to 84.070% (ninth). The TVE was 84.07%, exceeding the 60% threshold, thus acceptable [64-66,71,80-82]. If TVE falls below 60%, additional items are required to measure the constructs [65,66,71,80,81].

### About PCA

This study used PCA with varimax rotation to examine construct structure [65,66,71,82,88-90]. While 8 constructs were initially proposed, 9 components emerged, an acceptable variation in a new research setting that may reveal novel dimensions [64-66,81]. The organizational factors (O) construct was split into 2 components: component 4 (O6-O9) and component 7 (O2-O5), with O1 removed for a loading <0.6. Subsequently, the O construct was renamed ORG, consistent with the organizational domain in the HOT-Fit framework [13,30]. The other 7 constructs (SY, IQ, SQ, PEOU, PU, ITU, and UA) remained intact, with 41 of 42 items retained, preserving 8 constructs overall. These results demonstrate instrument stability with context-specific adaptation [10,12]. The items were then reorganized according to their revised constructs and components for subsequent data collection in the field study. PCA results are shown in Table S2 (Multimedia Appendix 8).

### The Instrument’s Internal Reliability

Internal reliability was assessed using Cronbach α [92], which evaluates item consistency in measuring underlying constructs [65,66,71,82,89,90]. A threshold of ≥0.7 was applied [93], and all constructs exceeded this value, confirming satisfactory reliability. PEOU showed the highest α (0.969), while PU was the lowest (0.914). These results demonstrate the strong strength of the measurement items. Cronbach α scores are detailed in Table S3 (Multimedia Appendix 8).

### Findings for the Field Study Through Measurement Model Assessment

The final measurement tool retained 41 items from the EFA. A minimum sample size of 300 was deemed adequate for CFA [71]; adjusting for a 20% nonresponse, the target was 375, with 343 participants completing the survey (91.5% response, no missing data) [78]. Unlike EFA, which is data-driven, CFA is theory-driven within SEM, confirming hypothesized factor structures through factor loadings and model fit indices [65,66,71,81,88]. CFA was used to validate the measurement model’s quality, reliability, unidimensionality, and validity [65,66,71,88,89]. Convergent validity was assessed via AVE, construct validity via model fit indices, and internal consistency via CR, preferred over Cronbach α [65,66,81,92]. Pooled-CFA which is demonstrated in Figure S1, in Multimedia Appendix 9 [66,88], was applied to evaluate all latent constructs simultaneously, increasing degrees of freedom and ensuring identification, even for constructs with fewer than 4 items [65,66,81]. This was suitable, as 1 construct contained only 2 items.

### Unidimensionality

Unidimensionality refers to a set of variables explained by a single construct [65,66,81]. It is achieved when all items for a construct show acceptable factor loadings, with low-loading items removed until fit indices are satisfied [65,66,71,81,84,88]. In this study, all items had loadings >0.6 (Table S1 in Multimedia Appendix 9), confirming unidimensionality [65,66,81].

### Validity and Normality Assessments

#### Convergent Validity

Convergent validity is the extent to which indicators measure a construct [65,66,71,81,94]. It is assessed through correlations among items and the AVE, where values >0.5 indicate adequacy [66,71,88,95]. All constructs in this study had AVE>0.5 (Table S1 in Multimedia Appendix 9), confirming convergent validity. ORG showed the highest AVE (0.857), and the environment component exhibits the lowest AVE (0.699); however, both are above the threshold.

#### Construct Validity

Construct validity was confirmed once model fit indices met recommended criteria [65,66,71]. This study applied RMSEA, CFI, TLI, and *χ*²_750_=1510.8 to assess absolute, incremental, and parsimonious fit, respectively [65,66,71]. The results showed RMSEA=0.054 (<0.08), CFI and TLI>0.90, and *χ*²/df=2.014 (<3.0), confirming construct validity (Table S2 in Multimedia Appendix 9).

#### Discriminant Validity

Discriminant validity was assessed to ensure that constructs were distinct and nonredundant. This was evaluated using the square root of AVE, which should exceed the construct’s correlations with other constructs [65,66,95]. As presented in Table S3 in Multimedia Appendix 9, all constructs met this criterion. The diagonal values (in bold) were higher than the corresponding row and column correlations, confirming satisfactory discriminant validity across the model [65,66,95].

#### About CR

CR was used to estimate the model’s internal consistency, with values between 0.6 and 0.7 considered acceptable [65,66,71]. As shown in Table S1 in Multimedia Appendix 9, all constructs exceeded this threshold. The ENV component had the lowest CR (0.903), while IQ recorded the highest (0.954), indicating satisfactory reliability across all constructs.

#### Normality Assessment

Normality was assessed by examining the skewness of each item. Skewness values between –1.5 and 1.5 are generally acceptable for normal distribution [66,71]. All constructs fell within this range, as shown in Table S1 in Multimedia Appendix 9, confirming that the data met the assumption of normality.

### Descriptive Statistics of Field Study

#### Response Rate

The questionnaire, which had been developed, was distributed to 375 respondents using proportionate stratified random sampling as part of the pilot study. The quantitative field study received 343 responses, achieving a 91.5% response rate. This number exceeds the minimum sample size of 100 recommended by Hair et al [71,80] and other scholars, making the current study suitable for analysis [65,66,81]. After thorough data cleaning and screening, all 343 responses were confirmed to be valid and free of missing data.

#### Demographic Statistics

Demographics were analyzed using categorical frequency (%) and numerical mean (SD). Respondents averaged 35.16 (SD 7.29) years, with 43/375, (41.7%) aged 31-40 years; 57.4% (197/375) were women. Medical officers formed 193/375 (56.3%), mostly with bachelor’s degrees 251/375 (73.2%). Most (207/375, 60.3%) had >5 years in MOH, averaging 9.7 (SD 7.27) years, while hospital tenure averaged 5.01 (SD 4.32) years, with (105/375, 30.6%) >5 years. Casemix training was reported by (241/375, 70.3%). Casemix knowledge (10 items, 10-point scale) showed (136/375, 39.6%) with a strong understanding, mean score of 71.78% (SD 17.59).

#### Descriptive Analysis of Each Construct

Descriptive analysis examined response distributions for all 41 items, including minimum, maximum, mean, and SD. For SY (4 items), mean scores ranged from 6.48-6.63 (average 6.56; SD 1.768-1.870). IQ (5 items) showed means 6.67-6.91 (average 6.80; SD 1.673-1.744). SQ had the highest means among independent variables, 6.94-7.55 (average 7.18; SD 1.537-1.761). ORG included structure (7.33-7.60, average 7.33; SD 1.614-1.817) and environment (6.69-6.98, average 6.85; SD 1.565-1.649). PEOU ranged 6.70-7.03 (average 6.84; SD 1.735-1.852). PU scored 6.91-7.25 (average 7.04; SD 1.814-1.920). ITU showed 6.80-7.12 (average 6.96; SD 1.689-1.797). The dependent variable UA recorded the strongest results, 7.12-7.63 (average 7.38; SD 1.589-1.682). Overall, results suggest high acceptance and positive perceptions across constructs (Table 1).

**Table 1:** The mean score and SD of all assessed constructs.

Item code	Mean (SD)	Average mean
SY1^a^	6.60 (1.768)	6.56
SY2	6.54 (1.839)	6.56
SY3	6.48 (1.870)	6.56
SY4	6.63 (1.827)	6.56
IQ1^b^	6.67 (1.679)	6.80
IQ2	6.85 (1.744)	6.80
IQ3	6.80 (1.673)	6.80
IQ4	6.77 (1.693)	6.80
IQ5	6.91 (1.688)	6.80
SQ1^c^	7.09 (1.613)	7.18
SQ2	6.94 (1.724)	7.18
SQ3	6.97 (1.537)	7.18
SQ4	7.34 (1.763)	7.18
SQ5	7.55 (1.761)	7.18
STR1^d^	7.21 (1.817)	7.33
STR2	7.33 (1.780)	7.33
STR3	7.60 (1.698)	7.33
STR4	7.17 (1.614)	7.33
ENV1^e^	6.98 (1.649)	6.85
ENV2	6.98 (1.585)	6.85
ENV3	6.69 (1.565)	6.85
ENV4	6.74 (1.618)	6.85
PEOU1^f^	6.73 (1.830)	6.84
PEOU2	6.70 (1.739)	6.84
PEOU3	6.76 (1.735)	6.84
PEOU4	7.03 (1.776)	6.84
PEOU5	7.01 (1.852)	6.84
PU1^g^	6.91 (1.841)	7.04
PU2	6.97 (1.911)	7.04
PU3	7.02 (1.814)	7.04
PU4	7.25 (1.920)	7.04
ITU1^h^	6.81 (1.704)	6.96
ITU2	7.01 (1.797)	6.96
ITU3	6.80 (1.689)	6.96
ITU4	7.07 (1.814)	6.96
ITU5	7.12 (1.773)	6.96
UA1^i^	7.29 (1.682)	7.37
UA2	7.12 (1.589)	7.37
UA3	7.21 (1.617)	7.37
UA4	7.63 (1.626)	7.37
UA5	7.63 (1.660)	7.37

^a^SY: system quality.

^b^IQ: information quality.

^c^SQ: service quality.

^d^STR: organizational structure.

^e^ENV: organizational environment.

^f^PEOU: perceived ease of use.

^g^PU: perceived usefulness.

^h^ITU: intention to use.

^i^UA: user acceptance.

#### Findings for Structural Model Assessment (CB-SEM) and Hypothesis Testing

After confirming unidimensionality, validity, and reliability through CFA, the latent constructs were mapped into the structural model for CB-SEM analysis [65,66,71]. The model included 8 constructs: SY, IQ, SQ, ORG, PEOU, PU, ITU (independent variables), and UA (dependent variable). CB-SEM assessed direct effects and tested hypotheses. Before CBSEM, CFA confirmed that all values satisfy the requisite thresholds for validity and reliability, thus the measurement models for all latent constructs within the model have been validated [65,66,81,84,93,96-102]. The final model was structured with exogenous constructs on the left, mediators in the center, and the endogenous construct (UA) on the right [65,66,81,82,88,90,97-99,103,104].

### Standardized Regression Estimates (Standardized Weights)

**Figure 2:**
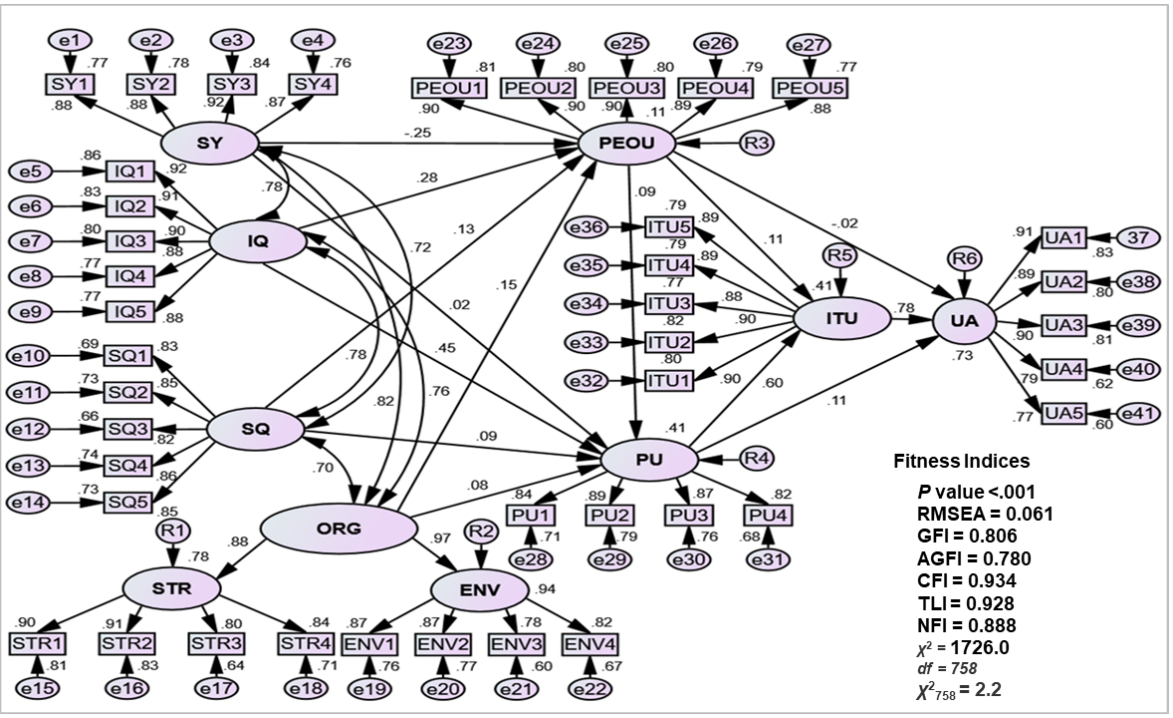
CB-SEM–standardized regression path coefficient between constructs. AGFI: adjusted goodness-of-fit index; CB-SEM: covariance based- structural equation modeling; CFI: Comparative Fit Index; ChiSq/df: chi-square/degree of freedom; e: measurement error; ENV: organizational environment; GFI: goodness-of-fit index; IQ: information quality; ITU: intention to use; NFI: Normed Fit Index; ORG: organizational characteristic; PEOU: perceived ease of use; PU: perceived usefulness; *R*^2^: squared multiple correlation; RMSEA: root-mean-square of error approximation; SQ: service quality; STR: organizational structure; SY: system quality; TLI: Tucker-Lewis Index; UA: user acceptance.

Table 2 exhibits the coefficient of multiple determination (*R*^2^) and implications. It was found that the squared multiple correlation (*R*^2^) for UA was 0.73 in the SEM inferential study of direct effects (Figure 2). This value accounts for up to 73% of all independent variables. In the Casemix-THIS context, the collaboration and mutual reliance on all 7 critical factors significantly facilitate medical doctors’ acceptance of the Casemix system. In the current IT environment, medical doctors find it challenging to embrace IT when only 1 or 2 factors support it. Using SPSS Amos (version 24.0), standardized regression analysis assessed correlations among the 4 exogenous constructs (SY, IQ, SQ, and ORG) and their effects on the endogenous construct (UA). The key outcome was the coefficient of determination (*R*²), indicating explained variance. Figure 2 illustrates causal paths (single-headed arrows) based on hypotheses and correlations among exogenous constructs (double-headed arrows) to avoid multicollinearity [65,66]. Table 2 shows that UA achieved an *R*² of 0.73, meaning 73% of its variance was explained by the 7 critical factors. In the Casemix-THIS context, this highlights that medical doctors’ acceptance of the Casemix system depends on the combined influence of all factors, as reliance on only 1 or 2 is insufficient in the current IT environment.

**Table 2:** The coefficient of multiple determination (*R*^2^^a^) and its implication.

Endogenous construct	*R* ^2^	Conclusion
PEOU^b^	0.11	SY^c^, IQ^d^, SQ^e^, ORG^f^, and PEOU manage to explain about 11% of the PEOU of the Casemix system implementation in THIS^g^ setting among medical doctors.
PU^h^	0.41	SY, IQ, SQ, ORG, and PEOU manage to explain about 41% of the PU of the Casemix system implementation in THIS setting among medical doctors.
ITU^i^	0.41	SY, IQ, SQ, ORG, PEOU, and PU manage to explain about 41% of the ITU of the Casemix system implementation in THIS setting among medical doctors.
UA^j^	0.73	SY, IQ, SQ, ORG, PEOU, PU, and ITU manage to explain about 73% of the UA of the Casemix system implementation in THIS setting among medical doctors.

^a^*R*^2^: squared multiple correlation.

^b^PEOU: perceived ease of use.

^c^SY: system quality.

^d^IQ: information quality.

^e^SQ: service quality.

^f^ORG: organizational characteristic.

^g^THIS: Total Hospital Information System.

^h^PU: perceived usefulness.

^i^ITU: intention to use.

^j^UA: user acceptance.

In order to avoid multicollinearity, correlations between exogenous constructs were assessed to evaluate the strength of the relationship between the exogenous constructs. A correlation above 0.85 indicates multicollinearity and a strong association [65,66,81,82,88,90,97-99,103,104]. As shown in Table 3, all 6 correlations were below this threshold, confirming no multicollinearity in the model [65,66].

**Table 3:** The correlation coefficient among exogenous constructs.

Constructs	Correlation coefficient	Description	Conclusion
SY^a^ and IQ^b^	0.78	SY and IQ play their roles in the acceptance of Casemix system implementation within THIS^c^ by 0.78	Discriminatory
SY and SQ^d^	0.72	SY and SQ play their roles in the acceptance of Casemix system implementation within THIS by 0.72	Discriminatory
SY and ORG^e^	0.82	SY and ORG play their roles in the acceptance of Casemix system implementation within THIS by 0.82	Discriminatory
IQ and SQ	0.78	IQ and SQ play their roles in the acceptance of Casemix system implementation within THIS by 0.78	Discriminatory
IQ and ORG	0.76	IQ and ORG play their roles in the acceptance of Casemix system implementation within THIS by 0.76	Discriminatory
SQ and ORG	0.70	SQ and ORG play their roles in the acceptance of Casemix system implementation within THIS by 0.70	Discriminatory

^a^SY: system quality.

^b^IQ: information quality.

^c^THIS: Total Hospital Information System.

^d^SQ: service quality.

^e^ORG: organizational characteristic.

### Unstandardized Structural Model of Regression Weights

**Figure 3:**
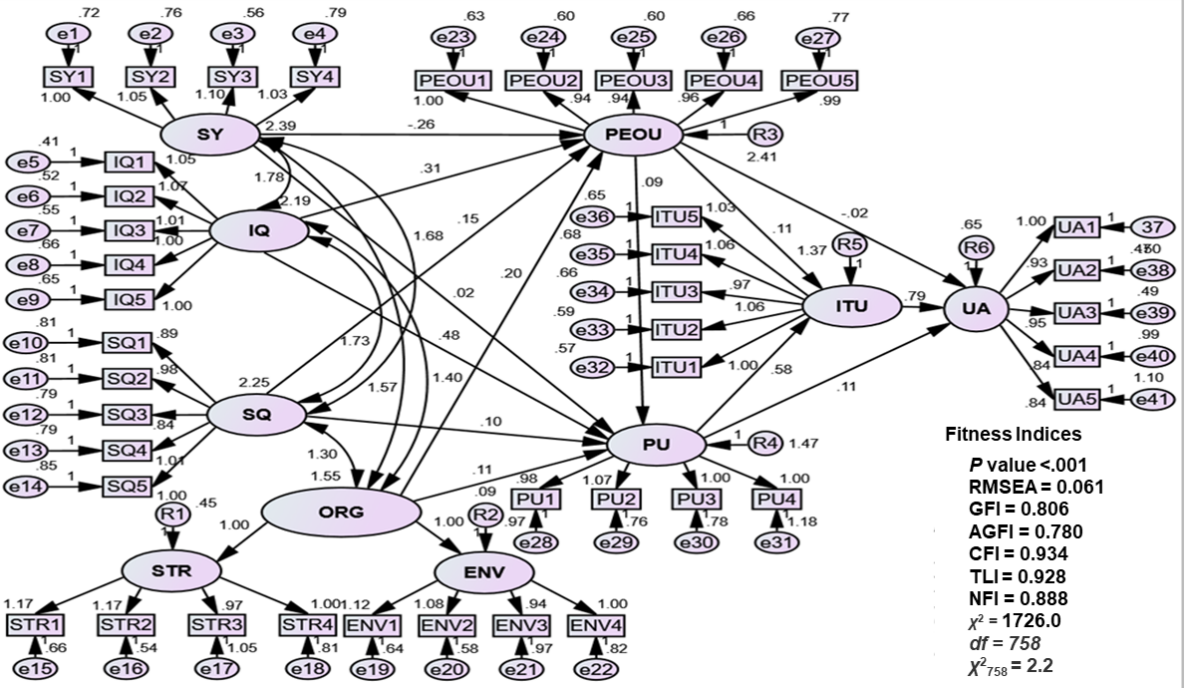
Model of regression weights (unstandardized structural model). AGFI: adjusted goodness-of-fit index; CFI: Comparative Fit Index; ChiSq/df: chi-square/degree of freedom; e: measurement error; ENV: organizational environment; GFI: goodness-of-fit index; IQ: information quality; ITU: intention to use; NFI: Normed Fit Index; ORG: organizational characteristic; PEOU: perceived ease of use; PU: perceived usefulness; *R*^2^: squared multiple correlation; RMSEA: root-mean-square of error approximation; SQ: service quality; STR: organizational structure; SY: system quality; TLI: Tucker-Lewis Index; UA: user acceptance.

The unstandardized regression model and path diagram (Figure 3) used double-headed arrows to connect all exogenous elements, as recommended in prior studies [65,66,81,82,85,90,93,105,106]. Single-headed arrows represented standardized regression weights (β coefficients). Most exogenous constructs showed positive effects on endogenous constructs, except for SY→PEOU (β=–0.262), indicating an inverse relationship where each unit increase in SY reduced PEOU by 0.262 units. This suggests potential usability or adaptation issues, aligning with prior studies on system design trade-offs. Regression path coefficients are shown in Table S1 (Multimedia Appendix 10), while Table S2 (Multimedia Appendix 10) summarizes the regression equations. Hypothesis testing was based on *P* values, with significance at *P*<.05 [84,85,93]. SPSS Amos (version 24.0) denoted *** for *P*<.001. Out of 8 hypothesized relationships (H1-H8), 5 were supported (H1, H2, H6, H7, and H8), while 3 were not (H3: SQ→PEOU, H4: ORG→PEOU, and H5: PEOU→PU). Table S3 (Multimedia Appendix 10) presents the regression path coefficients (β) from the exogenous constructs to the endogenous construct, as depicted in Figure 3. Table S4 (Multimedia Appendix 10) provides the corresponding regression equation derived from the standardized β estimates reported in Table S3 (Multimedia Appendix 10). Accordingly, Table 4 summarizes the detailed results.

**Table 4:** The regression path coefficients of the construct and its significance value.

H^a^	Exogenous		Endogenous	Standardized β estimate	SE	CR^b^	*P* value	Result
H1	SY^c^	→	PEOU^d^	–0.262	0.129	–2.028	.043	Significant
H2	IQ^e^	→	PEOU	0.307	0.125	2.455	.01	Significant
H3	SQ^f^	→	PEOU	0.146	0.111	1.318	.19	Not significant
H4	ORG^g^	→	PEOU	0.197	0.159	1.242	.21	Not significant
H5	PEOU	→	PU^h^	0.086	0.047	1.809	.07	Not significant
H6	PU	→	ITU^i^	0.580	0.053	10.962	<.001	Significant
H7	ITU	→	UA^j^	0.788	0.053	14.962	<.001	Significant
H8	PEOU	→	ITU	0.105	0.044	2.385	.02	Significant

^a^H: hypothesis.

^b^CR: critical ratio.

^c^SY: system quality.

^d^PEOU: perceived ease of use.

^e^IQ: information quality.

^f^SQ: service quality.

^g^ORG: organizational characteristic.

^h^PU: perceived usefulness.

^i^ITU: intention to use.

^j^UA: user acceptance.

### Quantitative Findings of Direct Path Hypothesis Analysis

The CB-SEM tested 8 direct path hypotheses, revealing a nuanced interplay between CSFs and Casemix system acceptance within the THIS context. Five hypotheses (H1, H2, H6, H7, and H8) were statistically supported, while 3 (H3, H4, and H5) were not, pointing to both expected and unexpected dynamics in health IT adoption. H1 showed a significant negative relationship between SY and PEOU (β=–0.262, *P*=.043), indicating that increasing system sophistication or technical complexity may inadvertently reduce usability, echoing concerns in TAM [39,56] and HOT-Fit [13,29,30,50] literature that advanced functionalities can burden end users if not aligned with clinical workflows. Conversely, H2 highlighted that IQ positively influenced PEOU (β=0.307, *P*=.01), consistent with the Information Systems Success Model (ISSM) [44,45], suggesting that reliable, accurate, and relevant information directly improves clinicians’ confidence and efficiency, thereby enhancing user experience.

However, SQ and ORG did not significantly influence PEOU (H3: β=0.146, *P*=.19; H4: β=0.197, *P*=.21), reinforcing the idea that such constructs exert indirect influence, primarily by creating an enabling environment rather than directly shaping doctors’ perceptions [13,29,30,60,107,108]. Similarly, H5, which tested the TAM pathway from PEOU to PU, was not supported (β=0.086, *P*=.07), implying that clinicians’ judgments of usefulness are driven more by whether the Casemix system enhances professional decision-making, accuracy, and reimbursement functionality than by ease of use alone [108,109]. However, PU emerged as a strong and significant determinant of ITU (H6: β=0.580, *P*<.001), affirming its central role in health IT adoption as consistently demonstrated in TAM and HIS studies [60,110]. ITU itself proved to be the most powerful predictor of UA (H7: β=0.788, *P*<.001), underscoring the behavioral dimension as the ultimate bridge between perceptions and actual uptake [42,108]. Finally, PEOU retained a modest yet significant influence on ITU (H8: β=0.105, *P*=.02), supporting the classical TAM assertion that usability enhances adoption likelihood, albeit as a secondary driver compared to PU [14,39].

Taken together, these findings highlight PU and ITU as the dominant forces driving Casemix acceptance, while SY and PEOU show more context-specific and sometimes paradoxical effects, reinforcing the importance of designing systems that balance technical sophistication with clinical usability. Moreover, the insignificance of SQ, ORG, and PEOU→PU suggests that adoption is less about infrastructural support or superficial ease, and more about the degree to which the system demonstrates clinical relevance, decision-support value, and alignment with reimbursement goals [60,108]. This not only confirms TAM’s robustness in health IT contexts but also points to the necessity of tailoring HIS implementation strategies to emphasize performance outcomes over usability alone.

### Categorization and Stratification of CSFs

This study identified and ranked the CSFs influencing UA of the Casemix system within THIS based on quantitative findings. Categorization was guided by statistical significance (*P* values) and standardized β estimates from the structural model. Four constructs emerged as significant predictors (*P*<.05): (1) ITU showed the strongest effect (β=0.788, *P*<.001), confirming its central role in technology adoption as proposed in TAM [14,42]. (2) PU ranked second (β=0.580, *P*<.001), highlighting adoption when users perceive efficiency and performance gains [14,42]. (3) IQ was third (β=0.307, *P*=.01), affirming that accurate, timely, and relevant information fosters trust and use, consistent with the DeLone and McLean Information Systems Success Model [45]. (4) SY showed a negative but significant effect (β=–0.262, *P*=.04), suggesting that usability constraints or technical issues may undermine acceptance despite functional robustness [45].

These 4 constructs are therefore the primary contributors to system acceptance. In contrast, 3 constructs were nonsignificant (*P*>.05) and ranked lower: (1) ORG (β=0.197, *P*=.21), reflecting leadership, training, and resources, may influence readiness indirectly but not acceptance directly. (2) SQ (β=0.146, *P*=.19), covering support responsiveness and reliability, lacked statistical significance despite theoretical relevance. (3) PEOU influenced ITU (β=0.105, *P*=.02) but not on PU (β=0.086, *P*=.07), limiting its classification as a primary factor.

Overall, ITU, PU, IQ, and SY represent the most influential factors and should be prioritized in system enhancement. ORG, SQ, and PEOU, while secondary, remain contextually relevant and should not be disregarded in complex health care implementation.

## Discussion

### Overview

This discussion section thoroughly examines the data, emphasizing the implications drawn from this study’s unique aims. It provides a comprehensive analysis of the critical success variables affecting the adoption of the Casemix system within the context of the THIS. This section thoroughly examines the direct effects of many study elements. This study offers a comprehensive analysis through direct effects, organized according to the research objectives. However, in this paper, the researchers emphasized the direct interrelationships among the 8 constructs by analyzing hypotheses H1 to H8. It aims to clarify the interaction of these aspects and their importance in facilitating the proper execution of the Casemix system in THIS context, while simultaneously answering the primary research issues.

### Principal Findings

### Findings of the EFA

The pilot study used EFA to identify underlying dimensions influencing Casemix-based THIS acceptance. Sampling adequacy was confirmed (KMO>0.6, BTOS *P*<.05), and factors with strong loadings (>0.6) were extracted following recommended practices [65,66,71,88,89]. The scree plot yielded 9 components across 42 items, with TVE of 84.07%, exceeding the 60% benchmark and indicating a robust factor structure [66,111], consistent with previous HIS adoption studies [29,38]. Compared to the 8 constructs of TAM and HOT-Fit [13,29,57], EFA revealed an additional demographic construct (socioeconomic and education) [66,111], and split organizational factors into structure (STR) and environment (ENV), aligning with HOT-Fit [13,29,30]. This supports evidence that organizational readiness and culture shape clinician engagement with Casemix [71]. The remaining constructs (SY, IQ, SQ, PEOU, PU, ITU, and UA) retained their integrity, aligning with TAM and ISSM findings that system and IQ drive satisfaction and adoption [14,45]. Reliability was strong (α=0.914-0.969) [66,92,112], comparable with prior HIS evaluations [66,87,113]. Overall, EFA validated 9 constructs for CFA, extending prior literature by integrating demographic influences and refining human, organizational, and technological dimensions. This underscores that successful HIT adoption requires both robust systems and supportive organizational-social contexts, providing a solid foundation for CFA and structural modeling [13,30,42].

### Findings of CFA

The CFA results confirmed the adequacy of the measurement model with strong fit indices (CFI=0.948, TLI=0.943, and RMSEA=0.054), aligning with theoretical expectations of HOT-Fit and TAM [13,14,29,30,42]. Similar to the study by Yusof et al [30], the emergence of ORG as a second-order construct with STR and ENV dimensions highlights the indirect role of leadership, resources, and culture in HIT adoption, consistent with the study by Yunus et al [38]. IQ showed high reliability (CR=0.954), supporting the importance of accuracy and timeliness for clinician trust [45,68]. PEOU demonstrated satisfactory loading despite a weaker influence, echoing TAM literature where mandated use reduces its effect compared to PU [14,42], a trend also noted in Casemix research [67,68]. Discriminant validity was established, reflecting the separateness yet interdependence of human, technological, and organizational dimensions [13]. Strong AVE (>0.5) and CR (>0.6) values reinforced construct clarity, consistent with validated adoption models [87,113,114]. These findings indicate that SY, IQ, and SQ integration remains crucial for clinician engagement [32], while PU and ITU continue as strong predictors of adoption [14,42]. Overall, the CFA supports the psychometric soundness of the instrument and extends evidence that Casemix acceptance in Malaysian THIS is shaped by the interplay of technology, organizational readiness, and user perceptions, providing a robust basis for subsequent structural modeling.

### Findings of the Structural Model

#### Overview

Following CFA validation of unidimensionality, reliability, and validity, the constructs were mapped into the SEM framework for hypothesis testing. The structural model comprised 8 latent constructs—SY, IQ, SQ, ORG, PEOU, PU, ITU, and UA, arranged from exogenous to endogenous variables to ensure logical flow. Seven independent constructs (SY, IQ, SQ, ORG, PEOU, PU, and ITU) were modeled to interact and ultimately influence the dependent construct, UA. Using CB-SEM, both standardized and nonstandardized regression paths were estimated, focusing on direct effects for hypothesis testing [14,29,30,42,65,66,71].

#### Standardized Weights for Standardized Regression Estimates

The SEM analysis revealed significant relationships between exogenous constructs, mediators, and the endogenous construct, UA. The squared multiple correlation (*R*²) for UA was 0.73, indicating that exogenous and mediating factors collectively explained 73% of its variance. This demonstrates the strong interdependence of constructs in influencing physicians’ adoption of the Casemix system. Moreover, all Pearson correlation coefficients among exogenous constructs were below the 0.85 cutoff, confirming discriminant validity and the absence of multicollinearity, which occurs when 2 exogenous constructs are substantially associated [65,66,82,90,98,103,104,115]. The correlation coefficients between exogenous components for 6 correlations, as shown in Table S2 in Multimedia Appendix 10, stayed below this cutoff, demonstrating that this model did not have any multicollinearity problems.

#### Unstandardized Regression Weights Structural Model

In the unstandardized regression model, double-headed arrows represented correlations between exogenous constructs, while single-headed arrows denoted causal paths, with standardized regression weights expressed as β coefficients. Most exogenous constructs positively influenced endogenous constructs; however, a negative path coefficient was observed between PEOU and SY (β=–0.262), indicating that higher SY reduced PEOU. This suggests usability trade-offs, consistent with prior findings on the balance between system complexity and user interaction [13,30,44-50].

#### Testing Hypotheses and Validating Models

Hypothesis testing used *P* values, with significance set at *P*<.05 [65,66,82,90,98,103,104,115]. Amos flagged results at *P*<.001. Of the 8 hypotheses (H1-H8), 5 were supported: H1 (SY→PEOU), H2 (IQ→PEOU), H6 (PU→ITU), H7 (ITU→UA), and H8 (PEOU→ITU). Three were not significant: H3 (SQ→PEOU), H4 (ORG→PEOU), and H5 (PEOU→PU). These results suggest that ITU, IQ, and SY significantly influence UA, while ORG and SQ have no direct effect on PEOU, and PEOU does not predict PU. Overall, SEM confirms that medical doctors’ acceptance of the Casemix system is primarily shaped by ITU, IQ, and SY, while anomalies in PEOU highlight potential usability trade-offs warranting further study.

### Discussion of The Quantitative Findings of Direct Path Hypothesis Analysis

The structural analysis examined direct effects among SY, IQ, SQ, ORG, PEOU, PU, ITU, and UA to understand Casemix system acceptance within THIS. Standardized regression results and hypothesis testing revealed nuanced relationships, highlighting key drivers of adoption and practical implications for system implementation.

A significant negative relationship was observed between SY and PEOU (H1: β=–0.262, *P*=.043), indicating that higher system functionality may increase complexity and reduce PEOU [39,56]. This finding aligns with TAM and HOT-Fit principles, which emphasize the need to balance technical performance with user-centered design [13,29,30,50]. High SY enhances reliability and confidence in system use [41,45], but interface complexity introduced by advanced features without phased training or iterative usability testing can negatively impact usability [108,116]. Vendors and hospital IT teams must therefore optimize system capabilities while maintaining intuitive navigation to support clinician adoption. IQ positively influenced PEOU (H2: β=0.307, *P*=.01), reinforcing its critical role in shaping usability perceptions and UA [45,49,52]. Accurate, complete, timely, and relevant information reduces cognitive burden and enhances satisfaction, confirming prior evidence that IQ is a central determinant of adoption in clinical settings [45,49,52].

SQ (H3: β=0.146, *P*=.19) and ORG (H4: β=0.197, *P*=.21) did not significantly affect PEOU, suggesting that in mature HIS environments, incremental improvements in service responsiveness or organizational support have limited influence on usability [29,30,108,116] Casemix’s standalone deployment and minimal integration reduce reliance on IT support or institutional facilitation, so clinicians may value system functionality and information accuracy over SQ or ORG [45,47,51,117-119]. While leadership, governance, and resource availability are known to facilitate system adoption [120,121], this study suggests that usability is judged primarily through direct system interactions, such as interface layout, navigation, and task alignment, rather than broader organizational policies or culture [119]. Similarly, PEOU did not significantly influence PU (H5: β=0.086, *P*=.07), highlighting that PU in complex clinical systems is shaped more by functional utility, system integration, and output relevance than by interface simplicity [39,108,122].

PU positively influenced ITU (H6: β=0.580, *P*<.001), indicating that users adopt systems they perceive as enhancing job performance, consistent with TAM and related frameworks [14,42]. Casemix’s ability to support efficient workflows, decision-making, and timely information increased ITU [45,123-125]. ITU strongly predicted UA (H7: β=0.788, *P*<.001), aligning with TAM and Unified Theory of Acceptance and Use of Technology models, which posit that behavioral intention is a robust predictor of actual system use [42]. This transition from ITU is especially critical in complex health care settings, where sustained adoption and integration are key to long-term system success [108]. A high ITU reflects strong user commitment, which in turn drives consistent engagement and fosters increased satisfaction [124]. Frequent interaction with the system can create a positive feedback loop, reinforcing both intention and actual usage over time [56]. PEOU had a significant but modest effect on ITU (H8: β=0.105, *P*=.02), suggesting that in hospital settings, ITU is more influenced by perceived utility and organizational support than by ease of use alone. These findings diverge from TAM, which posits that PEOU plays a crucial role in ITU [56]. Nonetheless, usability remains important, as intuitive systems reduce effort, enhance efficiency, and encourage regular engagement [45,123,124]. Training that improves PEOU can further strengthen ITU by boosting user confidence [123].

Collectively, these findings suggest 3 practical priorities for HIS or Casemix adoption. First, balancing functionality with usability is essential to prevent advanced SY from introducing cognitive overload, which can be mitigated through user-centered design, iterative usability testing, and phased feature deployment [108,126]. Second, prioritizing IQ, ensuring data accuracy, completeness, and timeliness, is critical to enhance both usability perceptions and clinical decision-making [45,127]. Finally, focusing on the functional value to drive adoption by demonstrating how Casemix improves performance, efficiency, and financial outcomes can strengthen ITU and UA, particularly in resource-constrained settings. By integrating these strategies into system design and implementation plans, the MOH can address the paradoxical effects observed in this study and enhance both acceptance and sustained use of Casemix within Malaysia’s hospital network.

### Discussion on Categorization and Ranking of CSFs

The categorization and ranking of CSFs highlight their varying influence on UA of the Casemix system, distinguishing high- from low-ranked predictors [42,45]. High-ranked CSFs (ITU, PU, IQ, and SY) showed significant effects (*P*<.05) and strong interrelationships, underscoring their central role in acceptance. These findings align with TAM, HOT-Fit, and ISSM, reaffirming the importance of behavioral intention, PU, and IQ in technology adoption [14,39,45]. Enhancing user motivation and system value perception thus remains critical for successful HIS implementation [30,108]. Lower-ranked CSFs (SQ, ORG, and PEOU), though theoretically relevant, showed limited direct effects. Their role may instead lie in enabling conditions, indirect pathways, or context-specific influences [29,30,128,129]. This suggests the need for institutional alignment and supportive environments to complement stronger determinants [130]. Overall, stratifying CSFs provides actionable priorities for stakeholders, focusing on high-impact constructs while recognizing the enabling role of lower-ranked factors. Future research should examine interaction effects and longitudinal dynamics to capture their evolving impact on sustained system adoption [116,131].

### Conclusion

This study advances understanding of the CSFs and determinants of medical doctors’ acceptance of the Casemix system within THIS of the MOH Malaysia. Using an integrated HOT-Fit and TAM framework, the ACT model was developed and empirically validated. The analysis revealed a clear stratification of CSFs into high-ranked and low-ranked predictors. High-ranked predictors, namely ITU, PU, IQ, and SY, emerged as the strongest drivers of adoption. These factors directly shape UA because they reflect clinicians’ beliefs in the system’s value, reliability, and ability to support both clinical and administrative tasks effectively. Enhancing these elements through improvements in data accuracy, system performance, and alignment of functionalities with clinical workflows will yield the greatest impact on adoption and sustained engagement. Low-ranked predictors, namely ORG, SQ, and PEOU, had weaker or nonsignificant effects. While still important, these enablers appear insufficient on their own unless paired with a robust, high-performing system that delivers tangible clinical and operational benefits.

From a policy and practice perspective, these findings inform 3 stakeholder-specific action pathways. For policy makers, it is essential to establish measurable Casemix adoption targets, integrate usage metrics into hospital performance indicators, enforce national data and interoperability standards, and allocate dedicated budgets for system upgrades to sustain high SY and IQ. For hospital administrators, visible leadership commitment to Casemix should be demonstrated, with usage linked to tangible clinical benefits, role-specific training provided to enhance PEOU, and daily workflows embedded with Casemix processes through supportive organizational policies. For system designers and vendors, system features should be codeveloped with clinicians to ensure clinical relevance. Interfaces should be streamlined to minimize cognitive load, and timely, accurate, and interoperable data outputs should be delivered to strengthen patient understanding and quality of care.

Beyond its local relevance, this research offers transferable lessons to other UMICs and LMICs where digital health adoption is challenged by resource constraints and fragmented systems. It demonstrates that functional performance, integration, and data quality are more decisive for acceptance than usability alone. This insight has direct implications for global HIS implementation strategies, particularly in environments where clinicians face heavy workloads and require systems that deliver measurable value from day one.

Finally, this study contributes to theory by refining the TAM and HOT-Fit models to reflect the realities of hospital-based HIS adoption and to practice by delivering a ranked, stakeholder-specific action plan. The findings support transitioning from the current standalone Casemix application (MyCMX 3.0) to an integrated Casemix-based HIS module embedded within THIS for all MOH hospitals. Such integration, coupled with standardized infrastructure, enforced vendor compliance, continuous training, and strong leadership engagement, will not only strengthen adoption but also ensure the Casemix system becomes a sustainable pillar of health care delivery. By acting on the strongest predictors first while progressively addressing secondary enablers, stakeholders can transform high intention into sustained real-world use, improving efficiency, resource allocation, transparency, and ultimately, the quality of care.

### Contributions or Implications

This study is one of the first to holistically examine the acceptance of Casemix system implementation within THIS among medical doctors in Malaysia, using an integrated model based on TAM and the HOT-Fit frameworks. It explores the influence of eight constructs, providing the theoretical, practical, social, and economic contributions.

### Theoretical Contributions

This study makes significant theoretical contributions to Casemix implementation in THIS hospitals under MOH Malaysia. The first is the development and empirical validation of the ACT model, integrating SY, IQ, SQ, ORG, PEOU, PU, ITU, and UA. Tested across 5 hospitals using CB-SEM, the model highlights the roles of SY, IQ, SQ, organizational support, and user perceptions in shaping UA, despite some nonsignificant direct paths (SQ and ORG on PEOU and PEOU on PU). The ACT model extends TAM and HOT-Fit frameworks, demonstrating applicability in UMICs and LMICs with varying HIS maturity. Its inclusion of context-specific constructs and validation through rigorous methods provides a replicable framework, addressing the gap where most digital health adoption studies focus on high-income countries [39,45,71,72,94,95,132]. Second, the current study develops a validated measurement instrument to assess Casemix acceptance in HIS contexts. Adapted from existing frameworks and refined via EFA and CFA, the 41-item tool is reliable for evaluating doctors’ readiness and perceptions. Its methodological rigor, combining pilot and field data with tests such as KMO, Bartlett, Cronbach α, and validity checks (unidimensionality, construct, convergent, and discriminant), ensures robustness [65,66,133-135]. Finally, methodologically, this study advances HIS research by using CB-SEM with pooled-CFA and second-order constructs, offering a replicable approach for complex systems such as Casemix. Its cross-sectional design further strengthens insights into acceptance and CSFs among practitioners in THIS hospitals [136,137].

### Practical Contributions

This study provides actionable insights to improve Casemix adoption and effectiveness within THIS under MOH Malaysia. First, embedding Casemix (Malaysian Diagnosis-Related Group version 2.0, MyCMX 3.0) into THIS, alongside CIS, NIS, LIS, RIS, PIS and PACS enables seamless data exchange, reduces duplication, and enhances real-time access for clinical, administrative, and financial tasks, improving usability, accuracy, and decision-making [32,33]. Second, policy support is critical. Aligned with the studies by Mukhriz et al [15] and Mukhriz and Gong [138], strategies include investing in digital infrastructure, user-friendly systems, standardized protocols, training, stakeholder engagement, privacy safeguards, and adoption incentives. Strong support systems (helpdesks and feedback loops) foster sustained use. Third, strengthening training is essential. Structured, role-specific programs improve confidence and ITU [41,42]. Train-the-trainer models, hands-on sessions, and varied learning tools (tutorials and manuals) reduce complexity. Fourth, user-centered design enhances PU and PEOU [139]. Features tailored to workflows, intuitive interfaces, phased rollouts, and performance tracking boost adoption. Fifth, demographic and role-based differences matter. Age, gender, tenure, and professional roles shape adoption [42]. Older and female users show stronger acceptance-intention links; senior professionals value relevance for clinical and financial decisions. Tailored communication and training support inclusivity. Finally, lessons for UMICs and LMICs emerge. Barriers such as fragmented workflows, limited interoperability, and weak support mirror other UMIC and LMIC experiences [140]. Usability alone is insufficient; integration, data quality, and functionality are stronger drivers. Malaysia’s Casemix experience offers guidance for integrating financing tools into HIS worldwide.

### Social Contributions

This study contributes socially by promoting equitable and efficient health care through Casemix implementation within THIS, identifying CSFs and determinants of acceptance among doctors. It highlights social readiness and usability concerns that shape health system reforms [108]. Supporting clinicians to adopt Casemix enables data-driven, consistent, and equitable care, improving access and SQ across hospital tiers [140]. Accurate coding also strengthens resource allocation, fair funding, and reflects true disease burdens [68,141-143]. Standardized classification enhances transparency, accountability, and trust in health care financing [144]. The current study underscores the importance of accurate clinical documentation, which improves patient histories, continuity of care, and personalized treatment [68,141-143], aligning with patient-centered care principles. By emphasizing SY, SQ, and PEOU, it calls for engaging providers through training and support to reduce resistance and foster ownership of digital reforms [145]. Such user-centric approaches ensure sustainable transitions in UMICs and LMICs such as Malaysia, where inclusivity and local relevance are critical [140]. In summary, the findings strengthen health care governance by aligning technology with social systems. By exploring how Casemix is perceived and adopted by frontline providers, this study supports Malaysia’s broader goal of Universal Health Coverage through informed decision-making, effective resource use, and socially responsive health systems [68,140-143].

### Economic Contributions

This study provides significant economic contributions by strengthening cost control and financial sustainability in public hospitals through better Casemix implementation within THIS. By identifying CSFs and UA elements influencing Casemix use among doctors, it supports more efficient coding and classification, leading to accurate reimbursement, optimized budgets, and reduced waste [146]. Casemix standardization of diagnoses and procedures enables performance-based financing and outcome-based budgeting, essential in publicly funded systems facing economic pressures for transparency and accountability [147]. Proper implementation ensures resource distribution matches service use and disease burden, improving planning and prioritization across departments [148]. SY, SQ, and PU further reduce inefficiencies such as delayed billing, miscoding, and duplication, which often cause revenue leakages and administrative costs [149]. By promoting accurate documentation, Casemix enhances rightful reimbursements from the central financing body [68,141-143]. Nationwide, the findings inform policies to expand Casemix use, supporting cost-effective health care through standardized data, economic evaluations, and benchmarking for better policy and investment strategies [140]. This aligns with Malaysia’s health financing reforms and sustainable health care goals under the Twelfth Malaysia Plan [150,151]. Worldwide, these insights support Universal Health Coverage and sustainable financing, as accurate Casemix coding improves resource allocation, cost control, and equity in service delivery, critical for health systems under financial constraints [152-154].

### Limitations

This study has several limitations. First, its quantitative design limited the depth of exploration, as qualitative methods could reveal additional CSFs, perceptions, and barriers. While quantitative evidence confirms predicted connections, qualitative insights can enrich understanding [155]. Second, reliance on self-reported questionnaires introduces potential biases (social desirability, recall, and acquiescence). Despite confidentiality assurances, such biases are inherent to survey-based research. Third, the cross-sectional design captures perceptions at a single point in time, restricting assessment of how acceptance evolves with prolonged use or policy changes. Longitudinal studies are recommended. Fourth, the focus on medical doctors and selected administrators in 5 MOH THIS hospitals enhances contextual depth but limits generalizability to other professionals, facilities without HIS, or different cultural settings. Comparative research across varied contexts would improve external validity. Fifth, no behavioral or usage metrics (eg, audit logs and task records) were available due to privacy and governance restrictions, limiting validation of whether intentions aligned with actual system use. Although self-reported measures are common in TAM and HOT-Fit studies, the absence of objective data may affect construct validity [14,29,42].

Sixth, similar limitations have been noted in other health IT adoption studies, where context-specific organizational, cultural, and governance factors significantly influence acceptance patterns, reducing applicability elsewhere. Adoption patterns may differ in private hospitals (market-driven priorities [156]), teaching hospitals (education-service missions [157]), or rural facilities (resource constraints [158]). Replication in international UMICs and LMICs with similar HIS goals would extend policy relevance, as health financing, governance, and information and communications technology capacity can alter adoption factors [159,160]. Such validation could refine the ACT model into a more universal HIS adoption framework by comparing high-ranked CSFs (eg, ITU, PU, IQ, and SY) with supportive factors (eg, ORG, SQ, and PEOU). Finally, the Malaysian public health care system has unique organizational and cultural characteristics that may not fully align with other UMICs and LMICs or high-income countries. While this limits transferability, the contextual insights contribute valuable evidence to the global discourse on digital health adoption, particularly in resource-constrained settings.

### Recommendations

This study offers insights for both global and Malaysian contexts. Worldwide, UMICs and LMICs face challenges such as fragmented IT infrastructure, inconsistent workflows, and reliance on standalone systems. Policy makers should standardize digital health components to ensure interoperability, data consistency, and scalability [23,161]. Phased integration of financing and classification tools such as Casemix into HIS, coupled with infrastructure upgrades, user-centered design, and structured training, is crucial for sustainable adoption [32,33]. For Malaysia, the priority is embedding Casemix (MyCMX 3.0) into THIS as a mandatory core component, supported by a standardized national framework across Basic Hospital Information System, IHIS, and THIS. A gradual transition should guide hospitals using manual or outdated HIS, with scalable web-based options maintained during the shift. Integration must align with health financing reforms to maximize clinical and administrative benefits.

Future research should adopt mixed methods to capture both quantitative and qualitative adoption dynamics [62]. Triangulation through observations, document analysis, and system logs can mitigate self-reporting bias and would offer a more comprehensive assessment [62,162]. Longitudinal designs are needed to track changes over time [163,164], and broader samples including nurses, IT staff, and administrators could reveal interdepartmental dynamics. Incorporating objective usage metrics (eg, health record audit log usage can reflect real-world workflows and support behavior inference) alongside surveys would strengthen validity, as prior studies show alignment between intention and actual use [165-167]. Further, subgroup and moderation analyses should examine how factors such as demographic profiles, education, working experience, tenure, and prior Casemix exposure shape adoption [108,168]. This would enable tailored training, resource allocation, and policy strategies. Locally, comparisons between public and private hospitals’ user exposure measures based on direct Casemix use, and extensions to the Ministry of Higher Education hospitals, would provide setting-specific insights [169,170].

In conclusion, these recommendations support the advancement of both global and local health information system adoption. Integrating Casemix into HIS, enhancing methodological approaches in research, and addressing organizational and user-level challenges will contribute to the development of efficient, equitable, and sustainable health care systems worldwide.

## Appendix

Multimedia Appendix 1 Study flowchart.

Multimedia Appendix 2 Ethics Approval of Medical Research of the Ethics Committee, MOH Malaysia, and the Faculty of Medicine, Universiti Kebangsaan Malaysia.

Multimedia Appendix 3 Operational definitions.

Multimedia Appendix 4 Inclusion and exclusion criteria flowchart.

Multimedia Appendix 5 Self-administered questionnaire.

Multimedia Appendix 6 Participant information sheet of quantitative study.

Multimedia Appendix 7 Consent form of quantitative study.

Multimedia Appendix 8 Exploratory factor analysis results.

Multimedia Appendix 9 Confirmatory factor analysis results.

Multimedia Appendix 10 Tables of co-variance based structural equation modelling.

## Abbreviations

ACT: Acceptance of Casemix in Total Hospital Information System
AVE: average variance explained
BTOS: Bartlett test of sphericity
CB-SEM: covariance-based structural equation modeling
CFA: confirmatory factor analysis
CFI: comparative fit index
CIS: clinical information system
CR: composite reliability
CSF: critical success factor
DRG: Diagnosis-Related Group
EFA: exploratory factor analysis
ENV: organizational environment
HIS: Hospital Information System
HIT: health care information technology
HOT-Fit: Human, Organization, and Technology Fit Evaluation Framework
IHIS: Intermediate Hospital Information System
INACBG: Indonesian Case-Based Groups
IQ: information quality
ISSM: Information Systems Success Model
ITU: intention to use
KMO: Kaiser-Meyer-Olkin
LIS: Laboratory Information System
LMIC: lower middle-income country
MalaysianDRG: Malaysian Diagnosis-Related Group
MOH: Ministry of Health
NIS: Nursing Information System
ORG: organizational characteristic
PACS: Picture Archiving and Communication System
PCA: principal component analysis
PEOU: perceived ease of use
PIS: Pharmacy Information System
PU: perceived usefulness
RIS: Radiology Information System
RMSEA: root-mean-square of error approximation
SQ: service quality
STR: organizational structure
SY: system quality
TAM: Technology Acceptance Model
THIS: Total Hospital Information System
TLI: Tucker-Lewis Index
TVE: total variance explained
UA: user acceptance
UMIC: upper middle-income country
