# Supplementary figures and images for: Critical Success Factors Influencing the Acceptance of a Casemix-Based Hospital Information System: Cross-Sectional Study
Source: J Med Internet Res. 2025 Sep 29;27:e74226. doi: 10.2196/74226 (PMC12533512; doi:10.2196/74226)

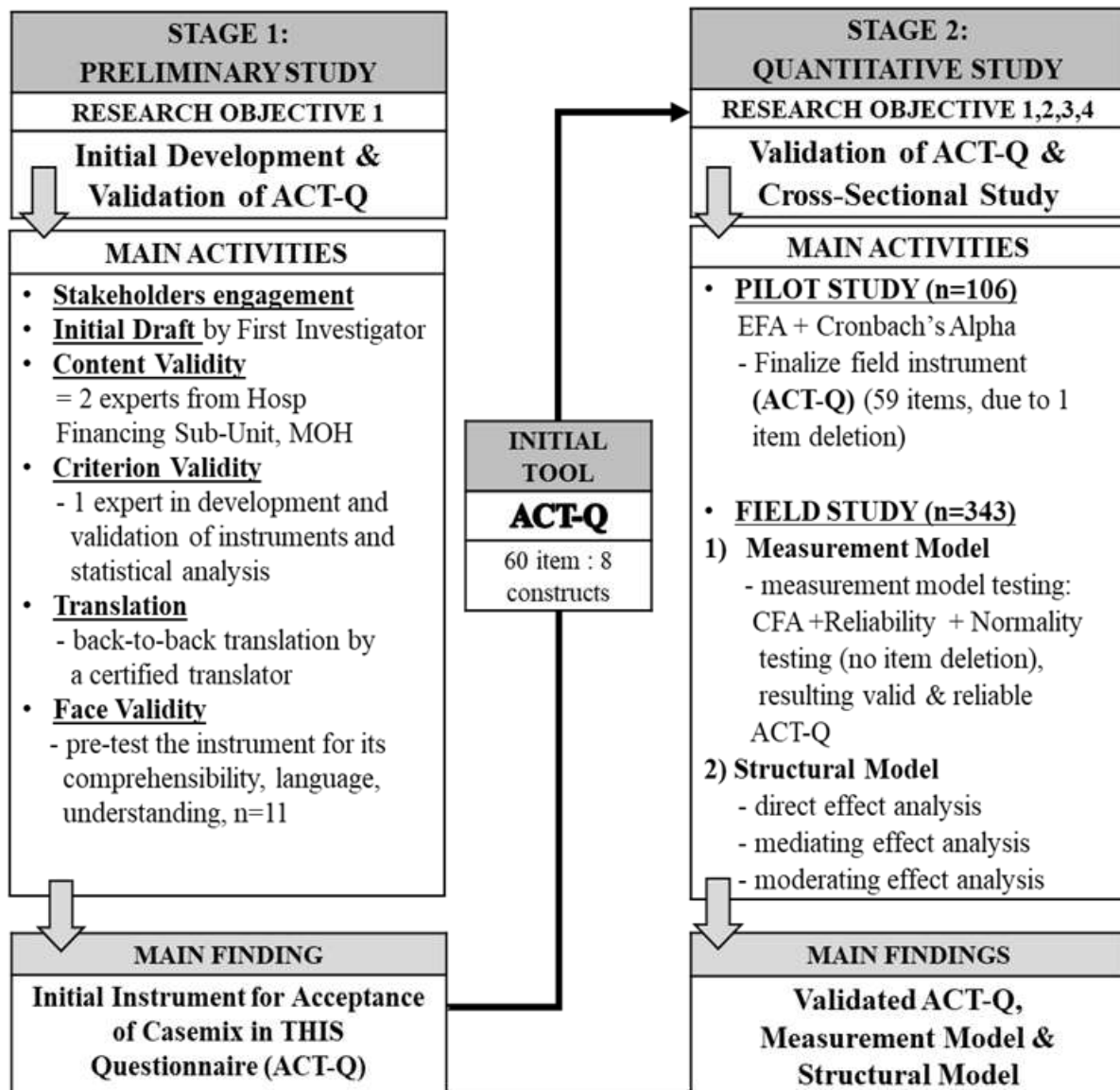

Figure S1. Flowchart for the cross-sectional study design procedure.

Supplement: Multimedia Appendix 1 [file jmir_v27i1e74226_app1.pdf]
