# Supplementary material for: Critical Success Factors Influencing the Acceptance of a Casemix-Based Hospital Information System: Cross-Sectional Study
Source: J Med Internet Res. 2025 Sep 29;27:e74226. doi: 10.2196/74226 (PMC12533512; doi:10.2196/74226)
Supplement: Multimedia Appendix 2 [file jmir_v27i1e74226_app2.pdf]

MEDICAL RESEARCH & ETHICS COMMITTEE MINISTRY OF HEALTH
MALAYSIA MINISTRY OF HEALTH MALAYSIA National
Institute of Health Complex (NIH) No. 1, Jalan Setia Murni U13/52,
Section U13 Bandar Setia Alam, 40170 Shah Alam, Selangor.

Tel.: +(6)03-33628888/ 33628205

Ruj. Kamil Ref: 22-02621-DKX Date/ Date:
2-02-2023

NOOR KHAIRIYAH BINTI MUSTAFA PUTRAJAYA
HOSPITAL

Dato'/ Dr/ Sir/Madam,

ETHICS APPROVAL LETTER/LETTER OF ETHICAL APPROVAL:

NMRR ID-22-02621-DKX (IIR)
CRITICAL SUCCESS FACTORS AND THE ACCEPTANCE OF CASEMIX SYSTEM IMPLEMENTATION IN TOTAL HOSPITAL INFORMATION SYSTEM OF THE MINISTRY OF HEALTH MALAYSIA

With respect the above is referred.

This letter is made in reference to the matter above.

2. Along with this letter is attached the letter of scientific and ethical approval for this project. All records and subject data are CONFIDENTIAL and only used for research purposes and all issues and procedures regarding data confidentiality must be followed. Permission from the Director of the Hospital / Institution where the study will be conducted must be obtained first before the study is conducted. Dato'/Mrs/Mrs need to agree and comply with the decision and other related laws.

*The Medical Research and Ethics Committee (MREC), Ministry of Health Malaysia (MOH) has provided ethical approval for this study. Please take note that all records and data are to be kept strictly **CONFIDENTIAL** and can only be used for the purpose of this study. All precautions are be taken to maintain data confidentiality. Permission from the District Health Officer / Hospital Administrator/ Hospital Director and all relevant heads of departments /units where the study will be carried out must be obtained prior to the study. You are required to follow and comply with their decision and all other relevant regulations.*

3. Researchers and research locations involved are:

The investigators and sites involved in this study are:

Putrajaya Hospital
Noor Khairiyah Binti Mustafa (Principal Investigator)

Sultanah Bahiyah Hospital, Alor Setar
Noor Khairiyah Binti Mustafa (Principal Investigator)

Sultan Ismail Hospital
Noor Khairiyah Binti Mustafa (Principal Investigator)

Sultanah Nur Zahirah Hospital, Kuala Terengganu Noor Khairiyah
Binti Mustafa (Principal Investigator)

Sarawak Heart Center Noor
Khairiyah Binti Mustafa (Principal Investigator)

4. The following study documents have been received and reviewed with reference to the above study:

The following study documents have been received and reviewed with reference to the above study:

Documents received and reviewed with reference to the above study: Documents received and reviewed with reference to the above study:

1. Cover letter to JEPP Cover letter to MREC (Version 1, dated 12-30-2022)

2. Declaration of Conflict of Interest Declaration of Conflict of Interest (COI) (Version 1, dated 12-30-2022)

3. Protocol Protocol (Version 1, dated 12-30-2022)

4. English Version: Patient Information Sheet & Informed Consent Form (Versi/ Version 1, dated 12-30-2022)

5. Malay Version: Patient Information Sheet & Informed Consent Form (Versi/ Version 1, dated 12-30-2022)

6. Data Collection Form Data Collection Form (Version 1, dated 12-30-2022)

7. English Version: Questionnaire (Version 1, dated 12-30-2022)

8. Malay Version: Questionnaire (Version 1, dated 12-30-2022)

9. Interview Guideline (Version 1, dated 12-30-2022)

10. Gantt Chart Gantt Chart (Version 1, dated 12-30-2022)

11. IA-HOD-IA, CV & GCP certificate/ certificate of GCP: Noor
    - Khairiyah Binti Mustafa

8. Be informed that this approval is valid until 01-02-2024. You need to send the following documents after getting ethical approval. The relevant forms can be downloaded from the National Medical Research Registry (NMRR) website.

   *Please note that the approval is valid until **01-02-2024**.The following are to be reported upon receiving ethical approval. Required forms can be obtained from the National Medical Research Registry (NMRR) website.*

   i. Continuing Review Form at the latest within 2 months (60 days) before the end of this approval period to renew ethics approval.

   ***Continuing Review Form** has to be submitted to MREC within 2 months (60 days) prior to the expiry of ethical approval.*

   i. Study Final Report at the end of the study to JEPP.

   ***Study Final Report** upon study completion to the MREC.*

   iii. Obtain ethical approval if there are amendments to any study document/study location/researcher. The JEPP has the right to withdraw ethical approval in the event of unannounced changes to study documents.

   *Ethical approval is required in the case of **amendments/ changes** to the **study documents/ study sites/ study team**. MREC reserves the right to withdraw ethical approval if changes to study documents are not completely declared.*

   iv. Clinical intervention studies only: Reports on all Serlous Adverse Events (SAEs), Suspected Unexpected Serious Adverse Reactions (SUSARs) and Protocol Deviation/Violation at the study site approved by JEPP if applicable. SAE must be reported within 15 calendar days from the awareness of the event by the researcher. The initial SUSAR report must be submitted as early as possible but not later than 7 calendar days from the researcher's awareness of the incident, followed by a complete report within an additional 8 calendar days.

   ***Applicable for Clinical interventional Studies only:** Report occurrences of **all Serious Adverse Events (SAEs), Suspected Unexpected Serious Adverse Reaction (SUSARs)** and **Protocol Deviation/Violation** at all MREC approved sites to MREC. SAEs are to be reported within 15 calendar days from awareness of event by investigator. Initial report of SUSARs are to be reported as soon as possible but not later than 7 calendar days from awareness of event by investigator, followed by a complete report within 8 additional calendar days.*

9. The number of subjects/patients/respondents who will be involved in this study in Malaysia is 452 people.

   *There will be **452 subjects/ patients/ respondents** involved in this study within Malaysia.*

10. Please be aware that any correspondence related to this research should mention the reference number of this letter to smooth related matters.

Please take note that the reference number of this letter must be stated in all future correspondence related to this study to facilitate the administrative processes.

The Medical Ethics & Research Committee, Ministry of Health Malaysia, operates according to the Council for Harmonization of Technical Requirements for Pharmaceuticals for Human Use (ICH). Any JEPP member involved in the evaluated study/project will not participate in the approval of the study/project.

The Medical Research & Ethics Committee, Ministry of Health Malaysia, operates in accordance to the International Council for Harmonization of Technical Requirements for Pharmaceuticals for Human Use (ICH). Any member of the MREC who is involved in the study/ project under review will not participate in the approval of the study/ project.

Study location/ Project Sites:
PUTRAJAYA HOSPITAL SULTANAH
BAHIYAH HOSPITAL, ALOR SETAR SULTAN ISMAIL
HOSPITAL
SULTANAH NUR ZAHIRAH HOSPITAL, KUALA TERENGGANU, SARAWAK
HEART CENTER

Decision by Medical Research & Ethics Committee: (√) Approved

() Did not pass/ Disapproved

Date of Ethical Approval: 02-02-2023

Thank you. Thank you.

I run the trust,

Van

....
Dr. NURAIN BINTI MOHD NOOR Chairperson
of the Medical Research &
Ethics Committee of the Ministry of Health Malaysia (No. MPM/ MMC No: 31576)

UNIVERSITI KEBANGSAAN MALAYSIA
The National University of Malaysia

CENTER FOR RESEARCH AND INSTRUMENTATION MANAGEMENT CENTER FOR RESEARCH AND INSTRUMENTATION MANAGEMENT

Reference: UKM PPI/111/8/JEP-2022-777
Date: January 13, 2023

Associate Major (PA) Dr. Roszita Ibrahim
Department of Community
Health Chancellor Tuanku Muhriz
Hospital UKM Medical Center

Y. Bhg. Professor/Datuk/Dato'/Datin/Sir/Madam,

ETHICS APPROVAL OF CONDUCTING RESEARCH AT UKM

Tajuk Penyelidikan : *Critical Success Factors And The Acceptance Of Casemix System Implementation In Total Hospital Information System Of The Ministry Of Health Malaysia*

The above is referred to.

2. Pleased to be informed, the UKM Research Ethics Committee approved the research application of Y. Bhg. Professor/Datuk/Dato'/Datin/Sir/Madam for the title above. The research approval period is from January 12, 2023 to January 11, 2025. Please submit any Side Effects Report, Progress Report Every 6 Months and Final Report as soon as the research is finished to the UKM Research Ethics Committee.

3. Please be reminded that this research project can only be carried out after receiving a letter of approval to carry out research from the Faculty's Deputy Dean of Research or Center/Institute Director.

Thank you.

Yours sincerely,

PROFESSOR DR. MOHD SHAHRIR MOHAMED SAID
Chairman
of the Research Ethics Committee of
the National University of Malaysia

s.k.

- Director of
  the Center for Research Management and Instrumentation (CRIM)
  of Universiti Kebangsaan Malaysia

- Director
  Tuanku Muhriz Chancellor Hospital,
  UKM Medical Center

- Deputy Dean (Research & Innovation)
  Secretariat of Medical Research & Innovation
  Chancellor Tuanku Muhriz
  Hospital UKM Medical Center

- **Ketua Jabatan Kesihatan Masyarakat**
  Hospital Canselor Tuanku Muhriz
  Pusat Perubatan UKM

- **Profesor Dato' Dr. Syed Mohamed Aljuni Syed Junid**
  Senior Public Health Medicine Consultant
  Professor of Health Economics
  Policy and Management Founding Chair Department of Health Policy and Management
  Faculty of Public Health
  Kuwait University

- **Profesor Madya Dr. Azimatun Noor Aizuddin**
- **Dr. Noor Khairiyah Mustafa (P115190 – Calon PhD)**
  Jabatan Kesihatan Masyarakat
  Hospital Canselor Tuanku Muhriz
  Pusat Perubatan UKM

- Fail Surat Kelulusan 2022

FI/FMZ

**NAME OF ETHICS COMMITTEE/IRB**:<br>Research Ethics Committee,<br>The National University of Malaysia	**ETHICS COMMITTEE/IRB REF NO :**<br>**UKM PPI/111/8/JEP-2022-777**
**PROTOCOL TITLE:**<br>Critical Success Factors And The Acceptance Of Casemix System Implementation In Total Hospital Information System Of The Ministry Of Health Malaysia	
**PRINCIPAL INVESTIGATOR:**<br>Mejar Bersekutu (PA) Dr. Roszita Ibrahim<br>Department of Community Heatlh<br>Hospital Canselor Tuanku Muhriz<br>UKM Medical Centre	

The following items [X] have been received and reviewed in connection with the above study to be conducted by the above investigator.

Documents

[X] Research Application Form
[X] Research Proposal / Protocol
[ ] Publication Policy
[X] Non-Disclosure Agreement
Information Sheet:-
[X] Malay [X] English
Consent Form:-
[X] Malay [X] English
Questionnaire:-
[X] Malay [X] English
Curriculum Vitae of Researcher:-
[X] Principal [X] Co-researcher [X] Student
[X] Good Clinical Practice Certificate (GCP)
[ ] Project Agreement

The Research Ethics Committee, The National University of Malaysia operates in accordance to the International Conference of Harmonization Good Clinical Practice Guidelines.

Comments (if any): Associate Professor Dr. Azimatun Noor Aizuddin is the co-investigator for this study and also member of Research Ethics Committee. She strictly was not involved in the decision of Research Ethics Committee to approve this study.

Date of Approval: 12 January 2023

PROFESSOR DR. MOHD SHAHRIR MOHAMED SAID
Chairman
Research Ethics Committee
The National University of Malaysia
