# Supplementary material for: Critical Success Factors Influencing the Acceptance of a Casemix-Based Hospital Information System: Cross-Sectional Study
Source: J Med Internet Res. 2025 Sep 29;27:e74226. doi: 10.2196/74226 (PMC12533512; doi:10.2196/74226)
Supplement: Multimedia Appendix 5 [file jmir_v27i1e74226_app5.pdf]

Multimedia Appendix 5. Self-administered questionnaire.

SELF-ADMINISTERED QUESTIONNAIRE
(BORANG KAJI SELIDIK)

Title: Critical Success Factors and Acceptance of Casemix System Implementation in The Total HospitalInformation System of The Ministry of Health Malaysia

Tajuk: Faktor-faktor Kejayaan Kritikal Dan Penerimaan Bagi Pelaksanaan Sistem Casemix di Sistem MaklumatHospital Menyeluruh Di Kementerian Kesihatan Malaysia

Instruction:

This questionnaire is divided into three sections which consist of i) Personal Details, ii) The Critical Success Factors of Casemix Implementation in THIS and iii) Outcome of the study which is the Acceptance Leveltowards Casemix System implementation in THIS setting. The purpose of this study is to examine the critical success factors and the acceptance of casemix System implementation in THIS facilities of the Ministryof Health (MoH) Malaysia among medical doctors in the hospital levels including the top management (medical doctors) as well as the clinicians.

Arahan:

Borang soalselidik ini terbahagi kepada tiga bahagian, iaitu ***i) Maklumat Peribadi, ii) Faktor Kejayaan Kritikalbagi Pelaksanaan Sistem Casemix di hospital yang dilengkapi fasiliti THIS dan iii) Hasil kajian iaitu Tahap Penerimaan terhadap Pelaksanaan Sistem Casemix di hospital yang dilengkapi fasiliti THIS.*** *Tujuan kajian iniadalah untuk mengkaji faktor-faktor kejayaan kritikal serta tahap penerimaan bagi Pelaksanaan Sistem Casemixdi hospital-hospital Kementerian Kesihatan Malaysia yang dilengkapi fasiliti THIS di kalangan para doctor di peringkat hospital yang terdiri daripada pengurusan tertinggi (dari kalangan doctor perubatan) serta para doktor klinikal.*

Please indicate your most appropriate response. All information and your chosen answers will be kept confidential. This study has also received ethical approval from the Medical Research Ethics Committee from the Ministry of Health Malaysia (NMRR-ID-22-02621-DKX) and from Universiti Kebangsaan Malaysia (JEP-2022- 777). For more information or clarification, do not hesitate to contact the Principal Researcher via her email address: p115190@siswa.ukm.edu.my or you can contact her via WhatsApp at 012-6161342.

Sila tandakan jawapan pilihan anda. Semua maklumat dan jawapan pilihan anda akan dirahsiakan. Kajian ini juga telah mendapat kelulusan etika daripada Jawatankuasa Etika Penyelidikan Perubatan dari Kementerian Kesihatan Malaysia ***(NMRR-ID-22-02621-DKX)*** *dan dari Universiti Kebangsaan Malaysia* ***(JEP-2022-777)****. Untuk maklumat lanjut atau penjelasan, jangan teragak-agak untuk menghubungi Penyelidik Utama melalui alamat e-mel beliau:* ***p115190@siswa.ukm.edu.my*** *atau anda boleh menghubunginya melalui WhatsApp di* ***012-6161342.***

Definition for Terms and Acronym *(Definisi Bagi Terma Dan Akronim):*

Here are some definitions of terms and acronyms that will be used in this questionnaire:

Berikut ialah beberapa definisi istilah dan akronim yang akan digunakan dalam soal selidik ini:

1) Casemix System: A system that **provides the healthcare industry with a consistent method of classifying types of patients, their treatment and associated costs**. It involves developing and implementing a patient classification system that groups patients according to their clinical conditions.
1) Sistem Casemix: *Sistem yang* ***menyediakan industri penjagaan kesihatan dengan kaedah yang konsisten bagi mengklasifikasikan jenis pesakit, rawatan mereka dan kos yang berkaitan****. Ia melibatkan pembangunan dan pelaksanaan sistem klasifikasi pesakit yang mengumpulkan pesakit mengikut keadaan klinikal mereka.*

***2)* Total Hospital Information System (THIS):** It is a project by the Ministry of Health (MOH) with the objective of providing a **complete ICT system** in establishing a **paperless** hospital environment in order to offer quality health services to the public-**an integration of clinical, administrative and financial systems.**
2) Total Hospital Information System (THIS)*: Ia adalah projek Kementerian Kesihatan (KKM) dengan objektif untuk menyediakan* ***sistem ICT yang lengkap*** *dalam mewujudkan* ***persekitaran hospital tanpa kertas*** *untuk menawarkan perkhidmatan kesihatan yang berkualiti kepada orang ramai—****satu integrasi sistem klinikal, pentadbiran dan kewangan****.*

***3)* MalaysianDRG Casemix System: A comprehensive system that records casemix data** (i.e demographic profiles, patient's encounter on arrival and admissions of patients, diagnosis, treatment, investigations and procedures, discharge numbers, patients' bed days) **from the hospitals to flow into the MOH data pool.**
3) MalaysianDRG Casemix System: Sistem komprehensif yang merekodkan data casemix *(iaitu* ***data demografik pesakit, data kedatangan dan kemasukan pesakit,*** *diagnosis, rawatan, penyiasatan dan prosedur, nombor pelepasan, hari tidur pesakit)* ***dari hospital untuk mengalir ke kumpulan data KKM.***

***4)* Sistem Maklumat Rawatan Pesakit (SMRP): A comprehensive medical treatment report system linkingall hospitals in the country.** The required data is entered manually (manual/ some BHIS/IHIS hospitals) or through integration from HIS (most THIS) and will be then integrated into MalaysianDRG Casemix System.
4) Sistem Maklumat Rawatan Pesakit (SMRP): Sistem laporan rawatan perubatan yang komprehensif menghubungkan semua hospital di negara ini*. Data yang diperlukan dimasukkan secara manual (manual/ beberapa hospital BHIS/IHIS) atau melalui integrasi daripada HIS (kebanyakan hospital THIS) dan kemudiannya akan disepadukan ke dalam Sistem MalaysianDRG Casemix*

Thank you for your cooperation.

Terima kasih atas kerjasama yang telah diberikan.

——

SECTIONS		
**SECTION 1A**<br><br>**Demographic Profile and Duration of Service**<br><br>***Profil Demografi dan Pengalaman Bekerja***		Please fill in/tick (√) your response in the appropriate boxes.<br><br>*Sila isikan/tandakan √ pada kotak jawapan pilihan anda.*
**1**	Gender<br>*Jantina*	□ Male (Lelaki)<br>□ Female (Wanita)
**2**	Age (in years)<br><br>*Umur (dalam tahun)*	____years (tahun)
**3**	Hospital<br><br>*Hospital*	□Hospital Putrajaya, WP Putrajaya<br><br>□Hospital Sultanah Nur Zahirah, Kuala Terengganu, Terengganu<br><br>□Hospital Sultan Ismail, Johor Bahru, Johor<br>□Sultanah Bahiyah, Alor Setar, Kedah<br><br>□Pusat Jantung Sarawak, Kota Samarahan, Sarawak
**4**	Professional Role or Position<br><br>*Peranan/Jawatan*	□ Hospital Director (Pengarah Hospital)<br>□ Deputy Director (Timbalan Pengarah)<br>□Consultant Specialist/Specialist (Pakar Perunding/Pakar)<br><br>□Medical Officer (Pegawai Perubatan)<br>□House Officer (Pegawai Perubatan Siswazah)
**5**	Education Background<br><br>*Latar Belakang Pendidikan Tertinggi*	□Post-Doctorate (Pasca-Doktor Falsafah)<br><br>□Philosophy Doctor (PhD)/Ijazah Doktor Falsafah<br>□Sub-Specialty (Sub-Kepakaran)<br><br>□Master's Degree (Ijazah Sarjana)<br>□Bachelor's Degree (Ijazah Sarjana Muda)
**6**	Duration of years working in Ministry of Health Malaysia (in years)<br><br>*Tempoh perkhidmatan dalam Kementerian Kesihatan Malaysia (dalam tahun)*	____years (tahun)
**7**	Duration of service at current hospital (in years)<br><br>*Tempoh perkhidmatan di hospital ini (dalamtahun)*	_____years (tahun)

**8**	Have you ever undergone training related to the Casemix system during your service at the Ministry of Health?<br><br>*Pernahkah anda menjalani latihan berkaitan*<br><br>*sistem Casemix sepanjang perkhidmatan anda di Kementerian Kesihatan?*	□ Yes (Ya) □No (Tidak)

<table>
<tr><td colspan="2">SECTION 1B
Knowledge on Casemix (Pengetahuan mengenaiCasemix)</td><td colspan="10">Please indicate your responses based on the scale from 1-10, with 1 being ‘no knowledge, and 10 being ‘Excellent Knowledge’

Sila tandakan jawapan pilihan anda mengikut skala dari 1-10, di mana 1 adalah ‘tiada

pengetahuan’, dan 10 adalah ‘pengetahuan yang sangat baik

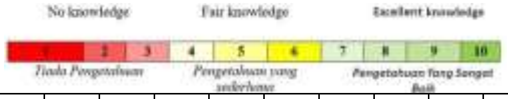
</td></tr>
<tr><td>1</td><td>Casemix system is one of the MOH's strategies to improve the quality of health care in medical and health facilities in Malaysia.

Sistem Casemix merupakan salah satu strategi KKM untuk menambahbaik kualiti penjagaan kesihatan difasiliti perubatan dan kesihatan di Malaysia.</td><td>1</td><td>2</td><td>3</td><td>4</td><td>5</td><td>6</td><td>7</td><td>8</td><td>9</td><td>10</td></tr>
<tr><td>2</td><td>The MalaysianDRG casemix system was introduced by the Ministry of Health (MOH) in October 2010 and subsequently implemented throughout the entire country.

Sistem casemix MalaysianDRG telah diperkenalkan oleh Kementerian Kesihatan (KKM) pada Oktober 2010 dan seterusnya dilaksanakan di seluruh negara.</td><td>1</td><td>2</td><td>3</td><td>4</td><td>5</td><td>6</td><td>7</td><td>8</td><td>9</td><td>10</td></tr>
</table>

**3**	The main objective of Casemix System is to estimate the costs that have been spent for each Diagnosis Related Group (DRG) or Main Diagnosis Category (MDC).<br><br>*Objektif utama pelaksanaan Sistem Casemix adalah untuk menganggar kos rawatan yang dibelanjakan ke atas sesuatu Diagnosis Related Group (DRG) or Main diagnosis Category (MDC).*	1	2	3	4	5	6	7	8	9	10
**4**	Casemix System involves and evaluates both clinical and costing data.<br><br>*Sistem Casemix melibatkan dan menilai kedua-duadata klinikal dan kewangan.*	1	2	3	4	5	6	7	8	9	10
**5**	Casemix System involves healthcare workers from various positions and disciplines.<br><br>*Sistem Casemix melibatkan kakitangan kesihatandaripada pelbagai jawatan dan disiplin.*	1	2	3	4	5	6	7	8	9	10
**6**	The implementation of the Casemix system that contains clinical data will involve demographic profiles, patient encounters on arrival and admissions of patients, diagnosis, treatment, investigations, and procedures.<br><br>*Pelaksanaan Sistem Casemix yang mengandungi data klinikal akan melibatkan data demografik pesakit, data kedatangan dan kemasukan pesakit,diagnosis, rawatan, ujian-ujian dan prosedur.*	1	2	3	4	5	6	7	8	9	10
**7**	The implementation of the Casemix system that contains financial data will involve discharge numbers, patients' bed days, encounter/workload for inpatients, percentage of time spent, hospital expenditure, building value, land value, outpatient and daycare, space areas, annual emolument, assets purchased, medical/health/dental supplies, out-of-pockets, and medical aid supports.<br><br>*Pelaksanaan sistem Casemix yang mengandungi data kewangan akan melibatkan bilangan discaj, hari tidur pesakit, pertemuan/beban kerja untuk*	1	2	3	4	5	6	7	8	9	10

	pesakit dalam, peratusan masa yang dihabiskan, perbelanjaan hospital, nilai bangunan, nilai tanah,pesakit luar dan jagaan harian, kawasan ruang, emolumen tahunan, aset yang dibeli, bekalan perubatan/ kesihatan/ pergigian, perbelanjaan luarpoket dan sokongan bantuan perubatan.										
**8**	The implementation of the Casemix System requires healthcare staff to provide accurate primary diagnosis, complete secondary diagnosis accurate main procedures, and a complete list of other procedures/ treatment/ investigations.<br><br>*Pelaksanaan Sistem Casemix memerlukan kakitangan penjagaan kesihatan untuk menyediakan diagnosis utama yang tepat, diagnosis lain yang lengkap dan prosedur utama yang tepat, dan senarai lengkap prosedur/rawatan/penyiasatan lain.*	1	2	3	4	5	6	7	8	9	10
**9**	Implementing Casemix System in the Total Hospital Information System (THIS) will integrate all required data into the Sistem Maklumat Rawatan Pesakit (SMRP), the MalaysianDRG Casemix System, and finally into the Executive Information System.<br><br>*Dengan pelaksanaan Sistem Casemix dalam Sistem Maklumat Hospital Menyeluruh (THIS), semua data yang diperlukan akan disepadukan ke dalam Sistem MaklumatRawatan Pesakit (SMRP), Sistem Casemix MalaysianDRG dan akhirnya ke dalam Executive Information System.*	1	2	3	4	5	6	7	8	9	10
**10**	Monitoring and evaluation is done through indicators that have been specially prepared for the Casemix System by the Malaysian Ministry of Health through the MalaysianDRG Casemix System application, as well as monitors through the audit function in the system as well as through audit documentation and codes on Per-PD 301.<br><br>*Pemantauan dan penilaian dilakukan*	1	2	3	4	5	6	7	8	9	10

	melalui indikator-indikator yang telah disediakan khas untuk Sistem Casemix oleh pihak Kementerian Kesihatan Malaysia melalui aplikasi MalaysianDRG Casemix System, serta pemantaun melalui fungsi audit dalam sistem serta melalui audit dokumentasi dan kod pada Per-PD 301										

**SECTION 2**<br>**Critical Success Factors (Faktor-faktor KejayaanKritikal)**		Please indicate your responses based on the scale from 1-10, with 1 being 'strongly disagree', *and 10 being 'strongly agree'*<br><br>*Sila tandakan jawapan pilihan anda mengikut skala dari 1-10, di mana 1 adalah 'sangat tidak setuju', dan 10 adalah 'sangat setuju'*<br><br>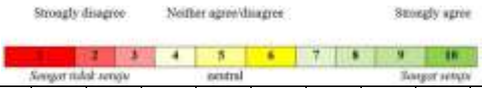									
**SY1**	The THIS and/or MalaysianDRG Casemix Systemare always available.<br><br>*THIS dan/atau MalaysianDRG Casemix Systemsentiasa tersedia.*	1	2	3	4	5	6	7	8	9	10
**SY2**	The THIS and/or MalaysianDRG Casemix Systemare user-friendly.<br><br>*THIS dan/atau MalaysianDRG Casemix System adalah mesra pengguna.*	1	2	3	4	5	6	7	8	9	10
**SY3**	The THIS and/or MalaysianDRG Casemix System provide interaction between users and the system.<br><br>*THIS dan/atau MalaysianDRG Casemix System menyediakan interaksi antara pengguna dan sistem.*	1	2	3	4	5	6	7	8	9	10
**SY4**	The THIS and/or MalaysianDRG Casemix System provides high-speed information access.<br><br>*THIS dan/atau MalaysianDRG Casemix System menyediakan akses maklumat berkelajuan tinggi.*	1	2	3	4	5	6	7	8	9	10

**IQ1**	The information required for the Casemix systemgenerated by THIS is accurate and correct.<br><br>*Maklumat yang diperlukan untuk Sistem Casemix dijana oleh THIS adalah tepat dan betul.*	1	2	3	4	5	6	7	8	9	10
**IQ2**	The information generated by the THIS is useful for Casemix purposes by providing complete information.<br><br>*Maklumat yang dijana oleh THIS berguna untuk*<br><br>*tujuan Casemix dengan menyediakan maklumatlengkap*	1	2	3	4	5	6	7	8	9	10
**IQ3**	The THIS generates information for Casemixpurposes promptly.<br><br>*THIS menjana maklumat untuk tujuan Casemixtepat pada masanya.*	1	2	3	4	5	6	7	8	9	10
**IQ4**	The information needed for Casemix is relevant all the time with THIS.<br><br>*Maklumat yang diperlukan untuk Casemix relevan sepanjang masa dengan THIS.*	1	2	3	4	5	6	7	8	9	10
**O4**	Hospital top management is responsible for providing training for Casemix and THIS.<br><br>*Pengurusan tertinggi hospital bertanggungjawab menyediakan latihan untuk Sistem Casemix dan THIS.*	1	2	3	4	5	6	7	8	9	10
**O5**	A series of training on the clinical documentation and costing module according to the stipulated guidelines prepared by the Ministry of Health Malaysia are adequate.<br><br>*Siri latihan dokumentasi klinikal dan modul kewangan mengikut garis panduan yang ditetapkan yang disediakan oleh Kementerian*	1	2	3	4	5	6	7	8	9	10

	Kesihatan Malaysia adalah mencukupi.										
**O6**	Organizational competency leads to the easiness of Casemix System adoption in THIS context reducing my workload.<br><br>*Kecekapan organisasi membawa kepada kemudahan penggunaan Sistem Casemix dalam konteks THIS mengurangkan beban kerja saya.*	1	2	3	4	5	6	7	8	9	10
**O7**	Supportive environment reduces user resistance to Casemix System adoption in THIS context.<br><br>*Kecekapan organisasi membawa kepada kegunaan penggunaan Sistem Casemix dalam konteks THIS.*	1	2	3	4	5	6	7	8	9	10
**O8**	The service providers of THIS and/or MalaysianDRG Casemix System adequately provide training to the users.<br><br>*Pembekal perkhidmatan THIS dan/atau MalaysianDRG Casemix System menyediakan latihan secukupnya kepada pengguna.*	1	2	3	4	5	6	7	8	9	10
**O9**	Adequate technical/application support is provided for Casemix System implementation in THIS context.<br><br>*Sokongan teknikal/aplikasi yang mencukupi*<br><br>*disediakan untuk pelaksanaan Sistem Casemix dalam konteks THIS.*	1	2	3	4	5	6	7	8	9	10
**PE1**	It is easy to find the information I needed from THIS and/or MalaysianDRG Casemix System.<br><br>*Mudah untuk mencari maklumat yang saya perlukan daripada THIS dan/atau MalaysianDRGCasemix System.*	1	2	3	4	5	6	7	8	9	10
	Using Casemix System with THIS is easy in all its steps.	1	2	3	4	5	6	7	8	9	10

**PE2**	*Menggunakan dan mengadaptasi Sistem Casemix dengan THIS adalah mudah dalam semua langkahnya.*										
**PE3**	I find it easy and flexible to get the THIS to do what I want for Casemix purposes.<br><br>*Saya rasa mudah dan fleksibel untuk THIS melakukan apa yang saya kehendaki bagi tujuan Casemix.*	1	2	3	4	5	6	7	8	9	10
**PE4**	It is easy to learn Casemix documentation with THIS support available.<br><br>*Sangat mudah untuk mempelajari penulisan dokumentasi klinikal Casemix dengan sokongan perkhidmatan THIS yang tersedia.*	1	2	3	4	5	6	7	8	9	10
**PE5**	My performance at work has improved as I amusing THIS and the Casemix System.<br><br>*Prestasi saya di tempat kerja telah bertambah baik kerana saya menggunakan THIS dan mengadaptasi Sistem Casemix*.	1	2	3	4	5	6	7	8	9	10
**PU1**	I am satisfied with the implementation of the Casemix System in THIS environment.<br><br>*Saya berpuas hati dengan pelaksanaan Sistem Casemix dalam persekitaran THIS.*	1	2	3	4	5	6	7	8	9	10
**PU2**	The use of the Casemix System in THIS environment has facilitated my job operations.<br><br>*Penggunaan Sistem Casemix dalam persekitaranTHIS telah memudahkan operasi kerja saya.*	1	2	3	4	5	6	7	8	9	10
**PU3**	Casemix System adoption in THIS context results in more success in achieving job objectives.<br><br>*Penerimaan Sistem Casemix dalam konteks THIS menghasilkan lebih*	1	2	3	4	5	6	7	8	9	10

	banyak kejayaan dalam mencapai objektif pekerjaan.										
PU4	I find the integration of THIS and/or SMRP and/orMalaysianDRG Casemix System useful in my job.<br><br>*Saya mendapati integrasi THIS dan /atau SMRP dan/atau MalaysianDRG Casemix System berguna dalam tugas saya.*	1	2	3	4	5	6	7	8	9	10
ITU1	I enjoy and am motivated to work in a THIS hospital where the Casemix System is being implemented.<br><br>*Saya suka dan bermotivasi untuk bekerja di hospital yang dilengkapi dengan THIS di mana Sistem Casemix sedang dilaksanakan.*	1	2	3	4	5	6	7	8	9	10
ITU2	I think the implementation of the Casemix System with THIS-provided services is a good idea.<br><br>*Saya merasakan pelaksanaan Sistem Casemix dengan perkhidmatan yang disediakan THIS adalah idea yang baik.*	1	2	3	4	5	6	7	8	9	10
ITU3	I believe the hospital staff is receptive to the implementation of the Casemix system.<br><br>*Saya percaya bahawa kakitangan hospital menerima pelaksanaan Sistem Casemix.*	1	2	3	4	5	6	7	8	9	10
ITU4	I agree with the idea of upgrading the facilities of THIS in other hospitals to implement Casemix System more effectively.<br><br>*Saya bersetuju dengan idea untuk menaik taraf dengan kemudahan THIS bagi hospital-hospital lain bagi melaksanakan Sistem Casemix dengan lebih berkesan.*	1	2	3	4	5	6	7	8	9	10
	I recommend using THIS to support CasemixSystem implementation.	1	2	3	4	5	6	7	8	9	10

**ITU5**	*Saya mengesyorkan penggunaan THIS bagi menyokong pelaksanaan Sistem Casemix.*										
	**SECTION 3**<br><br>**(User Acceptance)**	Please indicate your responses based on ascale from 1-10, with 1 being ‘strongly disagree’, *and 10 being ‘strongly agree’*<br><br>*Sila tandakan jawapan pilihan anda mengikut skala dari 1-10, di mana 1 adalah ‘sangat tidak setuju’, dan 10 adalah ‘sangat setuju’*<br><br>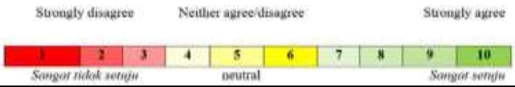									
**UA1**	Casemix System implementation with THISsetting facilitates easy access to patient information.<br><br>*Pelaksanaan Sistem Casemix dengan tetapan THIS memudahkan akses mudah kepada maklumat pesakit.*	1	2	3	4	5	6	7	8	9	10
**UA2**	Adoption of the Casemix System with THIS providedservice enables me to accomplish tasks more efficiently and improves the quality of my work.<br><br>*Pelaksanaan Sistem Casemix dalam tetapan THIS membolehkan saya menyelesaikan tugas dengan lebih cekap dan meningkatkan kualiti kerja saya.*	1	2	3	4	5	6	7	8	9	10
**UA3**	The implementation of the Casemix system inTHIS setting contributes to more accurate andcomplete diagnosis and procedures.<br><br>Pelaksanaan Sistem Casemix dalam tetapan THIS menyumbang kepada diagnosis dan prosedur yang lebih tepat dan lengkap.	1	2	3	4	5	6	7	8	9	10
**UA4**	The integration of Casemix System and THIS will help overcome the limitations of the paper-based system.<br><br>*Penyepaduan/Integrasi*	1	2	3	4	5	6	7	8	9	10

	MalaysianDRG Casemix System dan/atau THIS akan membantu mengatasibatasan sistem berasaskan kertas.										
**UA5**	Overall, I am satisfied with the Casemix System implementation in THIS setting.<br><br>*Secara keseluruhannya, saya berpuas hati dengan pelaksanaan Sistem Casemix dalam tetapan THIS.*	1	2	3	4	5	6	7	8	9	10
