# Supplementary material for: Critical Success Factors Influencing the Acceptance of a Casemix-Based Hospital Information System: Cross-Sectional Study
Source: J Med Internet Res. 2025 Sep 29;27:e74226. doi: 10.2196/74226 (PMC12533512; doi:10.2196/74226)
Supplement: Multimedia Appendix 6 [file jmir_v27i1e74226_app6.pdf]

Multimedia Appendix 6. Participant information sheet of quantitative study.

Title of study:
Critical Success Factors and The Acceptance of Casemix System Implementation in Total Hospital Information System of The Ministry of Health Malaysia

Name of investigators and institutions:

1. Dr. Noor Khairiyah binti Mustafa– Principal Investigator (UKM & KKM)
2. Assoc. Prof. Dr. Roszita binti Ibrahim – Main Supervisor (UKM)
3. Assoc. Prof. Dr. Azimatun Noor binti Aizuddin – Co-Supervisor (UKM)
4. Prof. Emeritus Dato' Dr. Syed Mohamed Aljunid bin Syed Junid – Co-Supervisor (IMU University)

Name of sponsor:
All will be self-funded by the principal investigator. This study does not receive sponsorship/funds from outside parties.

Introduction:
It is important that you understand why the research is being done and what it will involve.Please take your time to read through and consider this information carefully before you decide if you are willing to participate. Ask the study staff if anything is unclear or if you would like more information. After you are properly satisfied that you understand this study, and that you wish to participate, you must sign this informed consent form.

Your participation in this study is voluntary. You do not have to be in this study if you do not want to. You may also refuse to answer any questions you do not want to answer. If you volunteer to be in this study, you may withdraw from it at any time. If you withdraw, any data collected from you up to your withdrawal will still be used for the study. Your refusal to participate or withdrawal will not affect any medical or health benefits to whichyou are otherwise entitled.

This study was approved by both the Medical Research Ethics Committee from the Ministry of Health and the Faculty of Medicine, The National University Malaysia with thereference number: **NMRR ID-22-02621-DKX** and **JEP-2022-777** respectively.
What is the purpose of the study?
The purpose of this study is to evaluate the critical success factors and the acceptance of Casemix System implementation in the Total Hospital Information System of the Ministry of Health Malaysia.

This research will be conducted for 42 months from October 2021 till May 2025. However, the data collection period for the questionnaire might be done around February 2023till June 2023 once the ethics approval from MREC is obtained, starting with the pilot study. The expected number of participants is 125 for the pilot study and 375 individuals for the field study.

What are my responsibilities when taking part in this study?
It is important that you answer all the questions asked through the online questionnaire which is distributed by the study staff honestly and completely which will take about 10- 15 minutes of your time. Users also need to log in first with the e-mail address and it is easier to prevent duplicate entries from the same user. The investigators also did not measure the time people needed to fill in a questionnaire and excluded questionnaires that were submitted too soon. No timeframe was used as a cut-off point.

What are the details of the questionnaire?
The number of questionnaire items per page in Google Form is around 9-14 items, divided into 6 pages. It is technically possible to do consistency or completeness checks before the questionnaire is submitted. Was this done, and if "yes", an alternative is to check for completeness after the questionnaire has been submitted (and highlight mandatory items). If this has been done, it should be reported. All items did not provide a non-response option such as "not applicable" or "rather not say", but there are selections of one response option enforced.

What is the recruitment rate?
The recruitment rate is the respondents were able to review and change their answers through a Back button. The unique number of people who filled in the first survey page was counted (or agreed to participate by checking a checkbox), and divided by visitors who visited the first page of the survey (or the informed consent page, if present).

What is the completeness rate?
The completeness rate is the number of people submitting the last questionnaire page divided by the number of people who agreed to participate (or submitted the first survey page). There is a separate "informed consent" page or if the survey goes over several pages. The participants may leave questionnaire items blank.

What happens if I end the survey before its time?
If you decide to withdraw from the study midway, you could exit the Google Form freely and there are measures will be used to preserve the data you have filled in.
Only completed questionnaires will be analyzed. The questionnaires that terminated early (where, for example, users did not go through all questionnaire pages) were also analyzed, however, hence the missing data will be identified during analysis.
What are the potential risks and side effects of being in this study?
Participation in this study will not affect your treatment, and the risk is minimal. You arefree to decline to answer any of the questions that you feel uncomfortable with.

What are the benefits of being in this study?
There may or may not be any benefits to you. Information obtained from this study will help provides a foundation for Casemix System implementation and data integration in THIS hospitals. Moreover, the outcomes of this study can guide the stakeholders on howto adapt or improve Casemix system implementation in current contexts, as well as how to incorporate the Casemix system in various settings.

Who is funding the research?
All research funding will be borne by the main research student Dr Noor KhairiyahMustafa. You will also not be paid to participate in this study.
Will my medical information be kept private?
All your information obtained in this study will be kept and handled in a confidential manner, in accordance with applicable laws and/or regulations. When publishing or presenting the study results, your identity will not be revealed without your expressed consent. Individuals involved in this study, qualified monitors, and auditors, and governmental or regulatory authorities may inspect the study data, where appropriate andnecessary.

Who should I call if I have questions?
If you have any questions about the study or want information about this study, pleasecontact:

Principal Investigator
Dr. Noor Khairiyah binti Mustafa
Jabatan Perubatan Kesihatan Awam
Universiti Kebangsaan Malaysia
Tel: 012-6161342
Email: P115190@siswa.ukm.edu.my

For inquiries regarding the rights of the subject

Jawatankuasa Etika & Penyelidikan Perubatan
(Medical Research & Ethics Committee)
Kementerian Kesihatan Malaysia
Kompleks Institut Kesihatan Negara (NIH),
No.1, Jalan Setia Murni U13/52,
Seksyen U13 Bandar Setia Alam,
40170 Shah Alam, Selangor.
No.Tel: 03-33628407/33628205/ 33628888

Jawatankuasa Etika Penyelidikan Perubatan UKM
Sekretariat Etika Penyelidikan
Universiti Kebangsaan Malaysia,
Tingkat 1, Blok Klinikal,
Hospital Canselor Tuanku Muhriz,
Pusat Perubatan UKM,
Jalan Yaacob Latif,
Bandar Tun Razak,
56000 Cheras
Kuala Lumpur.
Tel: +603-9145 5046 / 9145 5048
E-mail: sepukm@ukm.edu.my

RISALAH MAKLUMAT PESERTA(BORANG KAJI SELIDIK)
(untuk subjek dewasa)

Tajuk Penyelidikan:
Critical Success Factors and The Acceptance of Casemix System Implementation in Total Hospital Information System of The Ministry of Health Malaysia
(Faktor Kejayaan Kritikal Dan Penerimaan Bagi Pelaksanaan Sistem Casemix di Sistem Maklumat Hospital Menyeluruh Di Kementerian Kesihatan Malaysia)

Nama penyelidik dan Nama Institusi:
1. Dr. Noor Khairiyah binti Mustafa– Penyelidik Utama (UKM & KKM)
2. Assoc. Prof. Dr. Roszita binti Ibrahim – Penyelia Utama (UKM)
3. Assoc. Prof. Dr. Azimatun Noor binti Aizuddin – Penyelia Bersama (UKM)
4. Prof. Emeritus Dato' Dr. Syed Mohamed Aljunid bin Syed Junid – Penyelia Bersama (IMU University)

Nama penaja:
Kajian ini tidak menerima penajaan/dana dari pihak luar. Semua pembiayaan kos kajian ditanggung oleh Penyelidik Utama.

Pengenalan:
Risalah ini menjelaskan hal-hal berkenaan penyelidikan tersebut dengan lebih mendalam dan terperinci. Amat penting anda memahami mengapa penyelidikan ini dilakukan dan apayang dilakukan dalam penyelidikan ini. Sila ambil masa yang secukupnya untuk membacadan mempertimbangkan dengan teliti penerangan yang diberi sebelum anda bersetuju untukmenyertai penyelidikan ini. Jika ada sebarang kemusykilan ataupun maklumat lanjut yanganda ingin tahu, anda boleh bertanya dengan mana-mana kakitangan yang terlibat dalam penyelidikan ini. Setelah anda berpuas hati bahawa anda memahami penyelidikan ini, dananda berminat untuk turut serta, anda dikehendaki untuk menandatangani Borang Persetujuan atau Keizinan Peserta, pada muka surat akhir risalah ini.

Penyertaan anda dalam penyelidikan ini adalah secara sukarela. Anda tidak perlu menyertaipenyelidikan ini jika anda tidak mahu. Anda juga mempunyai hak untuk tidak menjawab mana-mana soalan yang anda tidak mahu jawab. Anda juga boleh menarik diri daripada penyelidikan ini pada bila-bila masa sahaja. Jika anda menarik diri, segala maklumat yangtelah diperolehi sebelum anda menarik diri tetap akan digunakan dalam penyelidikan ini. Jika anda tidak mahu menyertai ataupun menarik diri dari penyelidikan ini, tindakan anda tidak akan menjejaskan segala hak dan keistimewaan perubatan kesihatan yang selayaknyaanda terima.

Kajian ini telah diluluskan oleh kedua-dua Jawatankuasa Etika Penyelidikan Perubatan dariKementerian Kesihatan dan Fakulti Perubatan, Universiti Kebangsaan Malaysia dengan nombor rujukan: NMRR ID-22-02621-DKX dan JEP-2022-777 masing-masing.

Apakah tujuan penyelidikan ini dilakukan?
Tujuan penyelidikan ini dilakukan adalah untuk mengenalpasti faktor-faktor kejayaan kritikal dan penerimaan bagi pelaksanaan Sistem Casemix dalam Sistem Maklumat Hospital Menyeluruh di Kementerian Kesihatan Malaysia.

Penyelidikan ini akan berlangsung selama 42 bulan dari Oktober 2021 hingga Mei 2025. Namun, proses pengumpulan data untuk fasa kuantitatif melalui borang kaji selidik dijangka bermula dari April 2023 hingga Jun 2023 selepas permohonan etika dan penyelidikan perubatan daripada MREC diperoleh. Dijangka bahawa 300 individu akan mengambil bahagian dalam kajian ini.

Apakah tanggungjawab saya sewaktu menyertai penyelidikan ini?
Amat penting anda menjawab kesemua soalan dalam borang kaji selidik dalam talian ini yang dikemukakan oleh kakitangan penyelidikan dengan jujur dan lengkap yang akan mengambil masa selama 10-15 minit. Pengguna juga perlu log masuk terlebih dahulu dengan alamat e-mel dan lebih mudah untuk mencegah entri duplikat daripada pengguna yang sama. Para penyelidik juga tidak mengukur masa yang diperlukan orang untuk mengisi soal selidik dan menolak soal selidik yang dihantar terlalu cepat. Tiada jangka masa digunakan sebagai titik pemotongan.

Apakah butiran soal selidik?
Bilangan item soal selidik setiap halaman dalam Google Form adalah sekitar 9-14 item, dibahagikan kepada 6 halaman. Secara teknikal, adalah mungkin untuk melakukan pemeriksaan konsistensi atau kelengkapan sebelum soal selidik dihantar. Adakah ini dilakukan, dan jika "ya", alternatifnya adalah untuk memeriksa kelengkapan selepas soal selidik dihantar (dan menyerlahkan item wajib). Jika ini telah dilakukan, ia harus dilaporkan. Semua item tidak menyediakan pilihan tidak memberi jawapan seperti "tidak berkenaan" atau "lebih suka tidak menjawab", tetapi terdapat pemilihan satu pilihan jawapan yang diwajibkan.

Apakah kadar perekrutan?
Kadar perekrutan adalah responden dapat menyemak dan menukar jawapan mereka melalui butang Kembali. Bilangan unik orang yang mengisi halaman soal selidik pertama dihitung (atau bersetuju untuk mengambil bahagian dengan menandakan kotak semak), dan dibahagikan dengan pelawat yang melawat halaman pertama soal selidik (atau halaman persetujuan dimaklumkan, jika ada).

Apakah kadar penyelesaian?
Kadar penyelesaian adalah bilangan orang yang menghantar halaman soal selidik terakhir dibahagikan dengan bilangan orang yang bersetuju untuk mengambil bahagian (atau menghantar halaman soal selidik pertama). Terdapat halaman "persetujuan dimaklumkan" yang berasingan atau jika soal selidik melalui beberapa halaman. Peserta boleh meninggalkan item soal selidik kosong.

Apa yang terjadi jika saya menamatkan soal selidik sebelum waktunya?
Jika anda memutuskan untuk menarik diri dari kajian di pertengahan, anda boleh keluar dari Google Form secara bebas dan terdapat langkah-langkah yang akan digunakan untuk memelihara data yang telah anda isi. Hanya soal selidik yang lengkap akan dianalisis. Soal selidik yang dihentikan awal (di mana, contohnya, pengguna tidak melalui semua halaman soal selidik) juga dianalisis, oleh itu data yang hilang akan dikenal pasti semasa analisis.

Apakah risiko dan kesan-kesan sampingan menyertai penyelidikan ini?
Risiko untuk penyertaan penyelidikan ini yang adalah minima. Anda berhak untuk tidak menjawab jika rasa tidak selesa dengan mana-mana soalan kajian.

Apakah manfaatnya saya menyertai kajian ini?
Penyelidikan ini mungkin akan mendatangkan manfaat ataupun langsung tiada memberi apa-apa manfaat kepada anda. Segala maklumat yang diperolehi daripada penyelidikan iniakan dapat membantu dalam mengenalpasti faktor-faktor kejayaan kritikal dan penerimaanbagi pelaksanaan Sistem Casemix dalam Sistem Maklumat Hospital Menyeluruh di Kementerian Kesihatan Malaysia.

Siapakah yang membiayai penyelidikan ini?
Segala pembiayaan penyelidikan akan ditanggung oleh pelajar penyelidik utama Dr Noor Khairiyah Mustafa. Anda juga tidak akan dibayar untuk menyertai kajian ini.
Adakah maklumat saya akan dirahsiakan?
Segala maklumat anda yang diperolehi dalam penyelidikan ini akan disimpan dan dikendalikan secara sulit, bersesuaian dengan peraturan-peraturan dan/ atau undang- undang yang berkenaan. Sekiranya hasil penyelidikan ini diterbitkan atau dibentangkan kepada orang ramai, identiti anda tidak akan didedahkan tanpa kebenaran anda terlebih dahulu.

Pihak-pihak tertentu seperti individu yang terlibat dalam penyelidikan ini, juruaudit dan jurupantau yang terlatih, pihak berkuasa kerajaan atau undang-undang, boleh memeriksa maklumat atau data kajian jika diperlukan.

Siapakah yang perlu saya hubungi sekiranya saya mempunyai sebarang pertanyaan?
Anda boleh menghubungi Penyelidik Utama kajian ini sekiranya anda mempunyai sebarang pertanyaan mengenai penyelidikan ini:

Penyelidik Utama
Dr. Noor Khairiyah binti Mustafa
Jabatan Perubatan Kesihatan Awam
Universiti Kebangsaan Malaysia
Tel: 012-6161342
Emel: p115190@siswa.ukm.edu.my

Jika anda mempunyai sebarang pertanyaan berkaitan dengan hak-hak anda sebagai peserta dalam penyelidikan ini, sila hubungi:

Jawatankuasa Etika & Penyelidikan Perubatan
(Medical Research & Ethics Committee)
Kementerian Kesihatan Malaysia
Kompleks Institut Kesihatan Negara (NIH),
No.1, Jalan Setia Murni U13/52,
Seksyen U13,
Bandar Setia Alam,
40170 Shah Alam,
Selangor.
No.Tel: 03-33628407/33628205/ 33628888

Jawatankuasa Etika Penyelidikan Perubatan UKM
Sekretariat Etika Penyelidikan
Universiti Kebangsaan Malaysia,
Tingkat 1, Blok Klinikal,
Hospital Canselor Tuanku Muhriz,
Pusat Perubatan UKM,
Jalan Yaacob Latif,
Bandar Tun Razak,
56000 Cheras,
Kuala Lumpur.
Tel: +603-9145 5046 / 9145 5048
E-mel: sepukm@ukm.edu.my
