# Supplementary material for: Critical Success Factors Influencing the Acceptance of a Casemix-Based Hospital Information System: Cross-Sectional Study
Source: J Med Internet Res. 2025 Sep 29;27:e74226. doi: 10.2196/74226 (PMC12533512; doi:10.2196/74226)
Supplement: Multimedia Appendix 8 [file jmir_v27i1e74226_app8.pdf]

Multimedia Appendix 8. Exploratory factor analysis results.

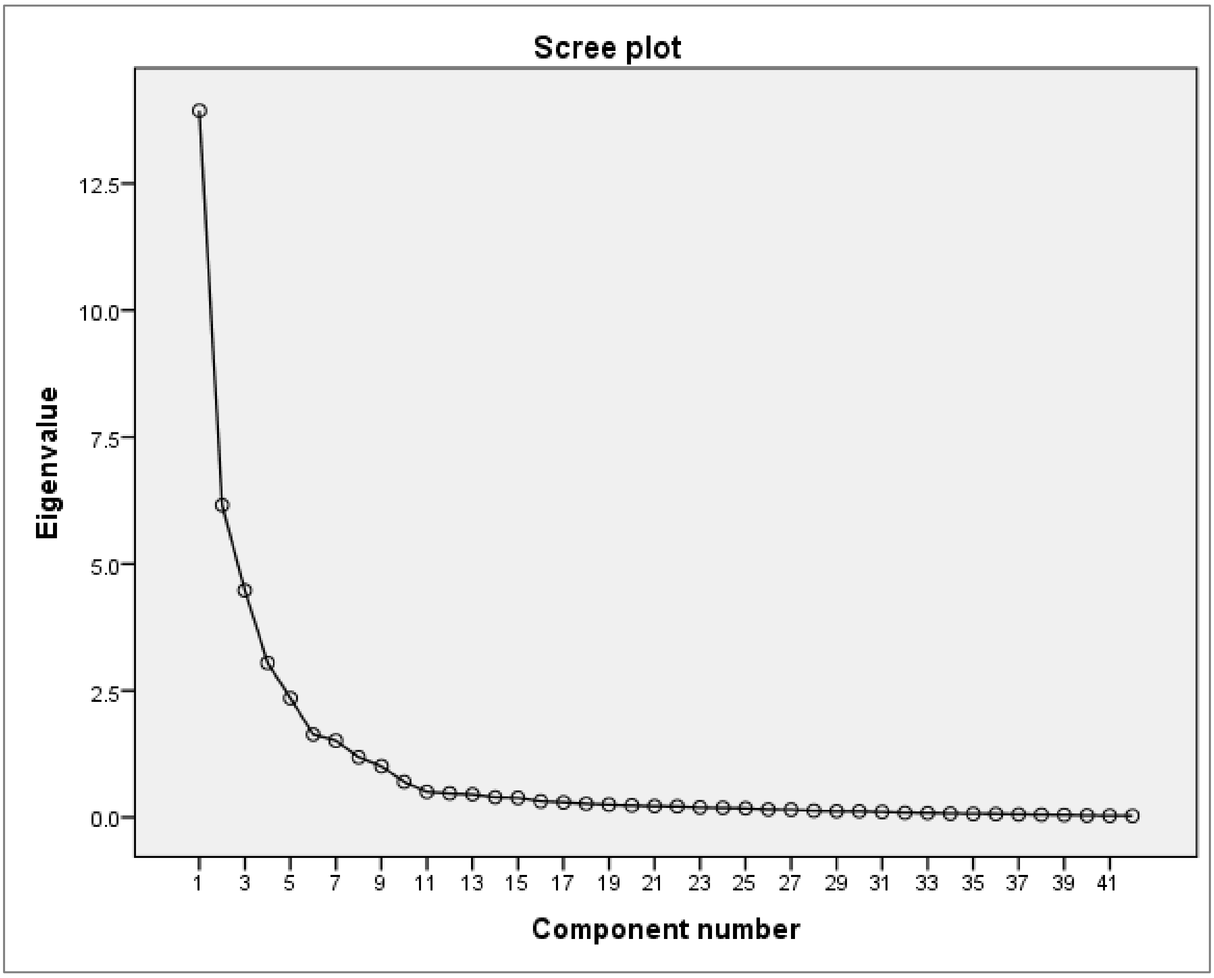

Figure S1. The scree plot indicating the 9 components that emerged for the constructs.

Table S1. The number of components formed and the amount of variance.

Component	Initial Eigenvalues<br>Total	 % of Variance	 Cumulative %	Rotation Sums of Squared Loadings<br>Total	 % of Variance	 Cumulative %
1	13.939	33.187	33.187	5.945	14.155	14.155
2	6.159	14.665	47.852	4.477	10.660	24.815
3	4.475	10.656	58.507	4.213	10.030	34.845
4	3.046	7.252	65.760	4.029	9.594	44.438
5	2.351	5.597	71.357	3.834	9.129	53.568
6	1.635	3.893	75.250	3.384	8.058	61.626
7	1.513	3.603	78.853	3.336	7.943	69.569
8	1.184	2.820	81.673	3.314	7.892	77.460
9	1.007	2.398	84.070	2.776	6.610	84.070

Table S2. Factor loading of EFA with PCA and varimax rotation.

	Rotated Component Matrix[a]								
	Component								
	1	2	3	4	5	6	7	8	9
PEOU1			0.908						
PEOU2			0.916						
PEOU3			0.889						
PEOU4			0.919						
PEOU5			0.895						
PU1								0.872	
PU2								0.888	
PU3								0.914	
PU4								0.872	
O1				~~0.590~~					
O2							0.873		
O3							0.746		
O4							0.874		
O5							0.861		
O6				0.827					
O7				0.864					
O8				0.808					
O9				0.882					
SY1					0.826				
SY2					0.821				
SY3					0.836				
SY4					0.853				
IQ1									0.614
IQ2									0.625
IQ3									0.654
IQ4									0.689
IQ5									0.619
SQ1		0.754							
SQ2		0.796							
SQ3		0.788							
SQ4		0.848							
SQ5		0.830							
ITU1	0.825								
ITU2	0.792								
ITU3	0.770								
ITU4	0.925								
ITU5	0.890								
UA1						0.676			
UA2						0.694			
UA3						0.625			
UA4						0.878			
UA5						0.833			

Extraction Method: Principal Component Analysis.
Rotation Method: Varimax with Kaiser Normalization.
[a] Rotation converged in 8 iterations.

Table S3. Cronbach α for each construct.

Construct	Name of Component	Items	No. of Items	Cronbach α (>0.7)
System Quality		SY1-SY5	5	0.968
Information Quality		IQ1-IQ4	4	0.950
Service Quality		SQ1-SQ5	5	0.902
Organizational Factors		O2-O9	8	0.933
	Structure	O2-O5	4	0.958
	Environment	O6-O9	4	0.919
Perceived Ease of Use		PEOU1-PEOU5	5	0.969
Perceived Usefulness		PU1-PU4	4	0.914
Intention to Use		ITU1-ITU5	5	0.949
User Acceptance		UA1-UA5	5	0.952
