# Supplementary material for: Critical Success Factors Influencing the Acceptance of a Casemix-Based Hospital Information System: Cross-Sectional Study
Source: J Med Internet Res. 2025 Sep 29;27:e74226. doi: 10.2196/74226 (PMC12533512; doi:10.2196/74226)
Supplement: Multimedia Appendix 9 [file jmir_v27i1e74226_app9.pdf]

Multimedia Appendix 9: Confirmatory factor analysis results.

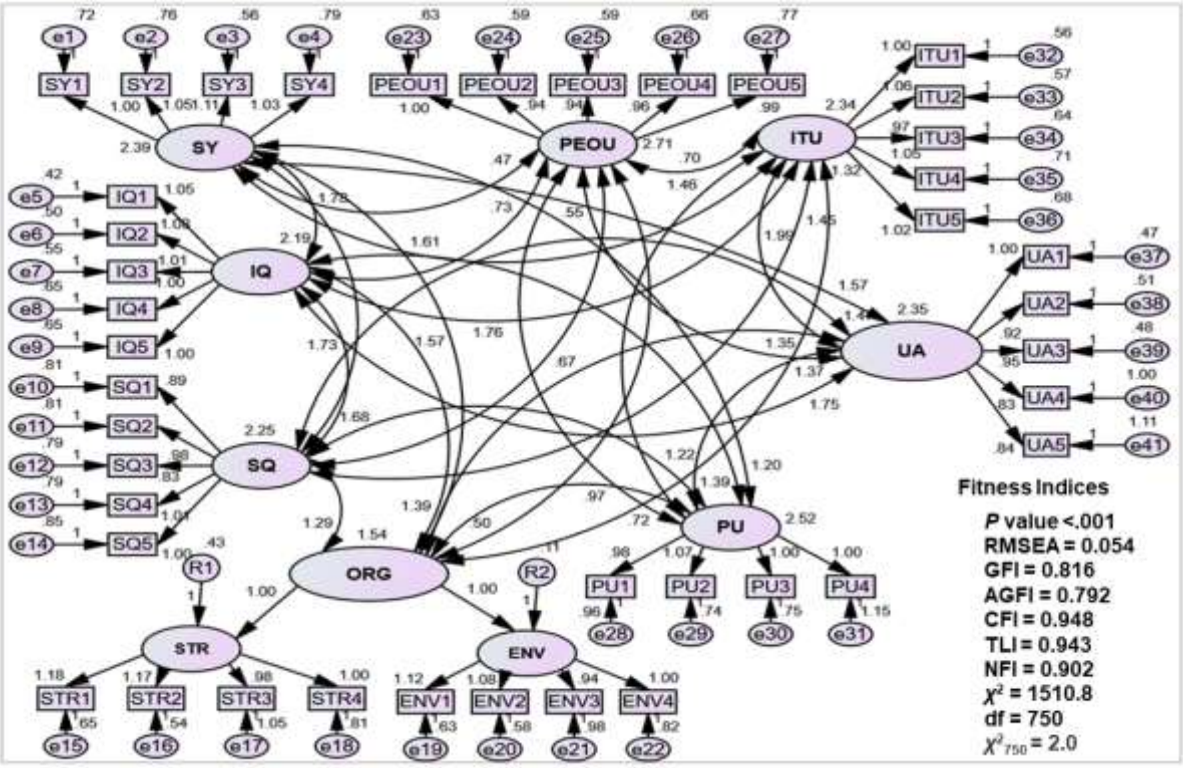

Figure S1. Pooled-confirmatory factor analysis.

Table S1. Factor loading of all items, composite reliability (CR), and average variance extracted (AVE) & normality testing.

**Construct**	**Component**	**Items**	**Factor Loading (>0.6)**	**CR (>0.6)**	**AVE (>0.5)**	**Skewness (-1.5 to 1.5)**
System Quality		SY1	0.876	0.936	0.787	-0.252
		SY2	0.880			-0.162
		SY3	0.917			-0.226
		SY4	0.873			-0.243
Information Quality		IQ1	0.923	0.954	0.806	-0.294
		IQ2	0.914			-0.055
		IQ3	0.895			-0.138
		IQ4	0.879			-0.018
		IQ5	0.877			-0.127
Service Quality		SQ1	0.829	0.925	0.710	-0.213
		SQ2	0.852			-0.233
		SQ3	0.815			-0.266
		SQ4	0.863			-0.175
		SQ5	0.852			-0.246
Organizational Characteristics	Structure		0.883	0.923	0.857	
	Environment		0.967			
	Structure	STR1	0.898	0.922	0.747	-0.390
		STR2	0.912			-0.227
		STR3	0.801			-0.284
		STR4	0.842			-0.109
	Environment	ENV1	0.875	0.903	0.699	-0.184
		ENV2	0.876			-0.315
		ENV3	0.773			-0.088
		ENV4	0.816			-0.107
Perceived Ease of Use		PEOU1	0.901	0.951	0.796	-0.267
		PEOU2	0.896			-0.314
		PEOU3	0.896			-0.360
		PEOU4	0.889			-0.313
		PEOU5	0.879			-0.284
Perceived Usefulness		PU1	0.845	0.920	0.742	-0.336
		PU2	0.893			-0.335
		PU3	0.879			-0.516
		PU4	0.828			-0.495
Intention to Use		ITU1	0.899	0.951	0.794	-0.107
		ITU2	0.907			-0.209
		ITU3	0.879			-0.325
		ITU4	0.884			-0.306
		ITU5	0.885			-0.331
User Acceptance		UA1	0.913	0.932	0.733	-0.532
		UA2	0.894			-0.542
		UA3	0.903			-0.441
		UA4	0.788			-0.395
		UA5	0.773			-0.413

Note: CR: composite reliability; AVE: average variance explained

Table S2. Fitness index summary.

Fitness category	Name of the Fitness Index	Full Name	Level of Acceptance	Index Value	Comment	Literature
Absolute fit	Chi-Square	Discrepancy Chi Square	*P*<0.05	<0.001	Not Applicable (Sample Size>200)	[1,2]
	RMSEA	Root mean Square of Error Approximation	< 0.1 (The best <0.08)	0.054	Achieved	[1,3,4]
	GFI	Goodness of Fit Index	>0.85 (The best >0.9)<br>(0.1-1.0)[a]	0.819	Achieved	[1,3,4]<br>[2][a]
Incremental fit	AGFI	Adjusted Goodness of Fit	>0.85 (The best >0.9)<br>(0.1-1.0)[a]	0.792	Achieved	[1,3,4]<br>[5][a]
	TLI	Tucker-Lewis Index	>0.85 (The best >0.9)	0.943	Achieved	[1,3,4]
	CFI	Comparative Fit Index	>0.85 (The best >0.9)	0.948	Achieved	[1,3,4]
	NFI	Normed Fit Index	>0.85 (The best >0.9)<br>(0.1-1.0)[a]	0.902	Achieved	[1,3,4]<br>[6][a]
Parsimonious Fit	Chi-Square/df	Chi-Square/Degree of Freedom	< 5.0 (The best <3.0)	2.014	Achieved	[1,3,4]

[a] : Other acceptable values and their related citations/literature.

Table S3. Discriminant validity index.

	SY	IQ	SQ	ORG	PEOU	PU	ITU	UA
System Quality (SY)	**0.887**							
Information Quality (IQ)	0.777	**0.898**						
Service Quality (SQ)	0.725	0.780	**0.842**					
Organizational Characteristics (ORG)	0.818	0.760	0.696	**0.926**				
Perceived Ease of Use (PEOU)	0.183	0.298	0.271	0.246	**0.892**			
Perceived Usefulness (PU)	0.488	0.592	0.511	0.493	0.274	**0.861**		
Intention to Use (ITU)	0.680	0.780	0.633	0.697	0.276	0.599	**0.891**	
User Acceptance (UA)	0.660	0.770	0.641	0.711	0.219	0.563	0.848	**0.856**

References:

1. Hair JF, Black WC, Babin BJ, Anderson RE, Black WC, Anderson RE. *Multivariate Data Analysis*.; 2019. doi:10.13106/jafeb.2021.vol8.no2.0943

2. Jöreskog KG, Sörbom D. *LISREL 8: User's Reference Guide (2nd Edition)*. Scientific Software International; 1996.

3. Awang Z. *Research Methodology and Data Analysis Second Edition*.; 2012.

4. Awang Z, Afthanorhan A, Lim SH, Zainudin NFS. *SEM Made Simple 2.0: A Gentle Approach of Structural Equation Modelling*. Universiti Sultan Zainal Abidin; 2023.

5. Tanaka JS, Huba GJ. A fit index for covariance structure models under arbitrary GLS estimation. *Br J Math Stat Psychol*. 1985;38(2):197-201. doi:10.1111/j.2044-8317.1985.tb00834.x

6. McDonald RP, Bollen KA. *Structural Equations with Latent Variables.* Vol 85.; 1990. doi:10.2307/2289630
