# Supplementary material for: Critical Success Factors Influencing the Acceptance of a Casemix-Based Hospital Information System: Cross-Sectional Study
Source: J Med Internet Res. 2025 Sep 29;27:e74226. doi: 10.2196/74226 (PMC12533512; doi:10.2196/74226)
Supplement: Multimedia Appendix 10 [file jmir_v27i1e74226_app10.pdf]

Multimedia Appendix 10: Tables of co-variance based structural equation modelling.

Table S1. The coefficient of multiple determination (R^2) and implication.

Endogenous Construct	R^2	Conclusion
Perceived Ease of Use	0.11	SY, IQ, SQ, ORG, and PEOU manage to explain about 11 percent of the PEOU of the Casemix system implementation in THIS setting among medical doctors.
Perceived Usefulness	0.41	SY, IQ, SQ, ORG, and PEOU manage to explain about 41 percent of the PU of the Casemix system implementation in THIS setting among medical doctors.
Intention to Use	0.41	SY, IQ, SQ, ORG, PEOU, and PU manage to explain about 41 percent of the ITU of the Casemix system implementation in THIS setting among medical doctors.
User Acceptance	0.73	SY, IQ, SQ, ORG, PEOU, PU, and ITU manage to explain about 73 percent of the UA of the Casemix system implementation in THIS setting among medical doctors.

Note: R^2: Squared multiple correlation; THIS: Total Hospital Information System; SY: System Quality; IQ: Information Quality; SQ: Service Quality; ORG: Organizational Characteristics; PEOU: Perceived Ease of Use; PU: Perceived Usefulness; ITU: Intention to Use; UA: User Acceptance

Table S2. The correlation coefficient among exogenous constructs.

Constructs	Correlation coefficient	Description	Conclusion
SY & IQ	0.78	SY and IQ play their roles in the acceptance of Casemix system implementation within THIS by 0.78	Discriminatory
SY & SQ	0.72	SY and SQ play their roles in the acceptance of Casemix system implementation within THIS by 0.72	Discriminatory
SY & ORG	0.82	SY and ORG play their roles in the acceptance of Casemix system implementation within THIS by 0.82	Discriminatory
IQ & SQ	0.78	IQ and SQ play their roles in the acceptance of Casemix system implementation within THIS by 0.78	Discriminatory
IQ & ORG	0.76	IQ and ORG play their roles in the acceptance of Casemix system implementation within THIS by 0.76	Discriminatory
SQ & ORG	0.70	SQ and ORG play their roles in the acceptance of Casemix system implementation within THIS by 0.70	Discriminatory

Note: THIS: Total Hospital Information System; SY: System Quality; IQ: Information Quality; SQ: Service Quality; ORG: Organizational Characteristics

Table S3. The regression path coefficient.

H	Exogenous	Endogenous	Std. Beta	Explanation
H1	SY	PEOU	-0.26	When SY goes up 1 unit, PEOU goes down by 0.26 units.
H2	IQ	PEOU	0.31	When IQ goes up 1 unit, PEOU goes up by 0.31 units.
H3	SQ	PEOU	0.15	When SQ goes up 1 unit, PEOU goes up by 0.15 units.
H4	ORG	PEOU	0.20	When ORG goes up 1 unit, PEOU goes up by 0.20 units.
H5	PEOU	PU	0.09	When PEOU goes up 1 unit, PU goes up by 0.09 units.
H6	PU	ITU	0.58	When PU goes up 1 unit, ITU goes up by 0.58 units.
H7	ITU	UA	0.79	When ITU goes up 1 unit, UA goes up by 0.79 units.
H8	PEOU	ITU	0.10	When PEOU goes up 1 unit, ITU goes up by 0.10 units.

Note: H: Hypothesis; Std: Standardized: SY: System Quality; IQ: Information Quality; SQ: Service Quality; ORG: Organizational Characteristics; PEOU: Perceived Ease of Use; PU: Perceived Usefulness; ITU: Intention to Use; UA: User Acceptance

Table S4. The regression equation for the model in this study.

Endogenous Construct		Regression Equation
Perceived Ease of Use	=	0.31 Information Quality - 0.26 System Quality + 0.15 Service Quality + 0.20 Organizational Characteristics
Perceived Usefulness	=	0.02 System Quality + 0.48 Information Quality + 0.10 Service Quality + 0.11 Organizational Characteristics + 0.09 Perceived Ease of Use
Intention to Use	=	0.10 Perceived Ease of Use + 0.58 Perceived Usefulness
User Acceptance	=	0.11 Perceived Usefulness + 0.79 Intention to Use – 0.02 Perceived Ease of Use
